# Differential Temperature-Induced Responses in Immortalized Oral and Skin Keratinocytes

**DOI:** 10.3390/ijms26072851

**Published:** 2025-03-21

**Authors:** Chen Han, Heidi Yuan, Amy K. Chen, Luisa A. DiPietro, Lin Chen

**Affiliations:** Center for Wound Healing and Tissue Regeneration, University of Illinois Chicago, Chicago, IL 60612, USA; chan206@uic.edu (C.H.); hyuan22@uic.edu (H.Y.); amychen1807@gmail.com (A.K.C.); ldipiet@uic.edu (L.A.D.)

**Keywords:** temperature-induced injury, skin, oral mucosa, keratinocyte, transcriptomics

## Abstract

The epidermis of the skin and oral mucosa is constantly exposed to various environmental stimuli, including temperature changes. In particularly extreme conditions, such as excess heat or cold, significant injury may occur. Oral and skin keratinocytes exhibit tissue-specific differences in wound healing outcomes and the transcriptomic response to injury. This study investigated if skin and oral keratinocytes also have differential responses to heat- and cold-induced injury. Oral keratinocytes (TIGKs) were found to exhibit an enhanced viability following heat-induced injury compared to skin keratinocytes (HaCaTs). However, there were no discernible differences between skin and oral keratinocyte viability following cold-induced injury. To examine the transcriptomic differences between skin and oral keratinocytes in response to temperature-induced injury, we generated an mRNA-sequencing gene expression dataset. Differentially expressed genes (DEGs) including heat shock proteins (HSPs) were identified between HaCaTs and TIGKs at baseline (37 °C) and after heat- (60 °C) or cold-induced (−25 °C) injury. Our comparative analyses suggest that skin and oral keratinocytes exhibit transcriptomic differences at baseline and in their responses to heat or cold exposure. The enhanced heat tolerance of TIGKs relative to HaCaTs may be due to an advantageous expression of a subset of HSPs at baseline in TIGKs. Our work also provides a source of skin and oral keratinocyte gene expression data following heat- and cold-induced injury that can be used for future analyses.

## 1. Introduction

The skin epidermis is the most superficial layer and provides an effective physical barrier that protects the body from various environmental stimuli. It is capable of withstanding constant exposure to altering environmental stimuli, such as temperature changes [1,2,3]. The skin’s response to hot and cold temperatures is a vital part of thermoregulation [3]. However, under extreme conditions, such as burns from excess heat or frostbite from excess cold, significant skin injury may occur [4,5]. The oral mucosa is another tissue that may be exposed to hot and cold temperatures, resulting in tissue injury. Generally, oral mucosa wounds heal more rapidly with less inflammation, faster re-epithelialization, and minimal scarring as compared to skin wounds [6,7,8,9,10,11,12,13,14,15]. One cell type that has been suggested to be responsible for conferring the differential responses of skin and oral mucosa to injury is keratinocytes [16,17,18,19].

Keratinocytes are the major cellular component of the epidermis of the skin and oral mucosa. They are crucial in the maintenance of epidermal homeostasis as they help to mediate the response to heat and cold exposure to the epidermis [3,20]. When heat or cold exposure begins to exceed normal cell thresholds, protective mechanisms are activated in skin and oral mucosa keratinocytes [4,5,20]. Since skin and oral keratinocytes exhibit intrinsic differences in response to physical injury [8], we speculate that they may also react differently to heat- and cold-induced injuries. Prior studies have demonstrated that both heat and cold shock can induce a stress response in keratinocytes characterized by the synthesis of stress proteins known as HSPs [5,20]. This stress response is evolutionarily conserved and may prevent cell death following heat- and cold-induced injury [20]. It is unknown if skin or oral mucosa keratinocytes have differential tolerance to heat and cold.

Our study assesses whether heat- and cold-induced injury may elicit similar transcriptomic responses in skin or oral mucosa keratinocytes. Our study found that oral keratinocytes have an enhanced tolerance to heat as compared to skin keratinocytes. Our findings suggest that the differential heat tolerance of skin and oral keratinocytes may be due to the transcriptomic differences that exist between them at baseline and after heat- or cold-induced injury. Overall, our work suggests that skin and oral keratinocytes have distinct responses to temperature-induced injury.

## 2. Results

### 2.1. TIGKs Exhibit Enhanced Resistance to Heat Injury

**Figure 1:**
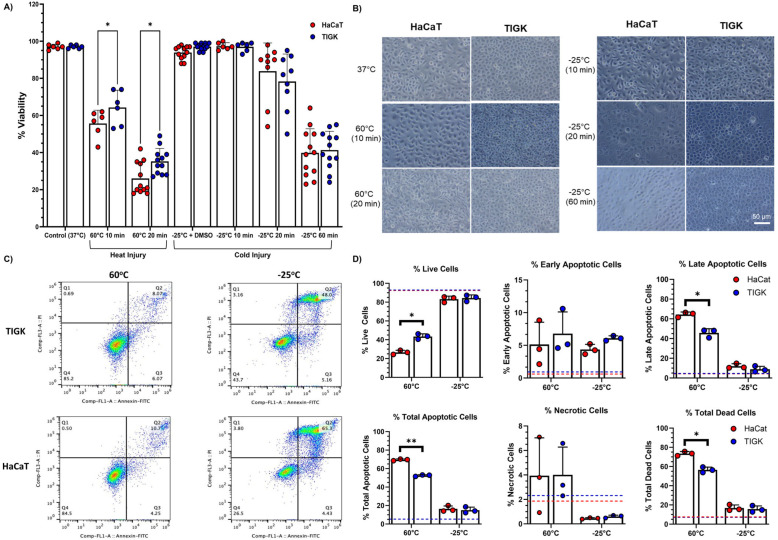
Oral keratinocytes are more tolerant to heat-induced injury than skin keratinocytes. (**A**) Viability of oral keratinocytes (TIGKs) and skin keratinocytes (HaCaTs) under hot and cold conditions. N = 6–12. Data shown are pooled from 2–4 repeated experiments. (**B**) Representative photos of HaCaTs and TIGKs exposed to hot and cold conditions. (**C**,**D**) HaCaTs and TIGKs were harvested after being exposed to 60 °C or −25 °C for 10 min. Cell death assessment was performed using an Annexin V Apoptosis Detection kit analyzed by flow cytometry. Representative images of flow cytometry analysis. Scale bar = 50 μm. (**C**). Q1: necrotic cells, Q2: late apoptotic cells, Q3: early apoptotic cells, Q4: live cells. Summary of cell death assessment (**D**). N = 3. Baseline values for HaCaTs and TIGKs are shown in the dashed red and blue lines, respectively. The data shown is representative of 2 experiments. * = *p* < 0.05, ** = *p* < 0.01. Two-way ANOVA was used for A and Multiple *t*-test was used for D.

To assess whether there were differences in the heat and cold tolerance of skin and oral keratinocytes, HaCaTs and TIGKs were subject to heat- or cold-inducing injury conditions, and cell viability was assessed. Initially, for our heat-inducing injury conditions, we assessed cell viability after HaCaTs and TIGKs were exposed to 50 °C for 20 min, but did not observe significant cell death. For these reasons, our heat-inducing injury conditions utilized 60 °C temperatures for varying amounts of time. A temperature of at least 60 °C is known to guarantee keratinocyte cell death [4,21,22]. Cold-inducing injury conditions utilized −25 °C temperatures for varying amounts of time. Both heat- and cold-induced injury resulted in significant cell death for HaCaTs and TIGKs (Figure 1A). Compared to HaCaTs, TIGKs exhibited significantly higher viability after 10 or 20 min heat-induced injury (Figure 1A). There were no differences between HaCaTs and TIGKs viability after 10, 20, or 60 min cold-induced injury (Figure 1A). There were no observable morphological changes in either HaCaTs or TIGKs following heat-induced injury (Figure 1B). However, after cold-induced injury, TIGKs became smaller (Figure 1B). When frozen with DMSO-containing media, HaCaTs and TIGKs did not show decreased viability nor any morphological changes when compared to their own respective 37 °C baseline control. Furthermore, live, apoptotic, and necrotic cells were also identified in HaCaTs and TIGKs exposed to 60 °C or −25 °C for 10 min using an annexin V apoptosis detection kit. HaCaTs had significantly more late/total apoptotic and total dead cells than TIGKs following heat-inducing injury conditions (Figure 1C,D). Thus, TIGKs had significantly more live cells than HaCaTs (Figure 1C,D). There were no differences observed in viability between HaCaTs and TIGKs after cold-induced injury conditions (Figure 1C,D).

### 2.2. HaCaTs and TIGKs Cluster Both by Cell Type and Injury

**Figure 2:**
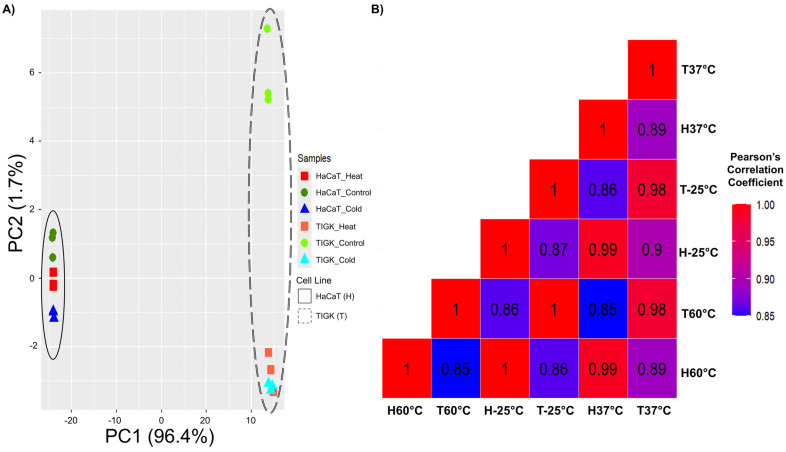
Comparative analysis of the transcriptomic response of oral and skin keratinocytes to injury reveals they cluster by cell type and temperature-induced injury. (**A**) Principal component analysis plot of mRNA-sequencing expression data. Each sample is represented by a colored point on the graph. The x-axis and y-axis are the first and second principal components, respectively. (**B**) Heatmap representing similarities of the mean gene expression profiles grouped by keratinocyte cell type (oral vs skin) and temperature condition: −25 °C, 37 °C, and 60 °C. Each square and its color represents aggregate Pearson’s correlation coefficient values between each experimental group (cell type and temperature condition).

In vitro gene expression datasets were generated through mRNA-sequencing analysis of HaCaTs and TIGKs cultured at 37 °C or after being exposed to 60 °C or −25 °C for 10 min. The same time of exposure for cell death evaluation by flow cytometry was utilized so that valid comparisons could be made. PCA showed that the first 2 PCs account for 96.4% and 1.7% of the observed variance in the dataset, respectively, and distinguish the samples by cell type and temperature exposure (Figure 2A). Samples were then grouped by cell type and temperature exposure, and the mean gene expression value was calculated for each gene within each group. Pearson’s correlation was calculated for each group’s mean gene expression data as well (Figure 2B). Together, our results show that HaCaTs and TIGKs cluster first by cell type and then by their exposure to hot or cold temperatures (Figure 2).

### 2.3. HaCaTs and TIGKs Exhibit Transcriptomic Differences at Baseline and in Response to Temperature-Induced Injury

**Figure 3:**
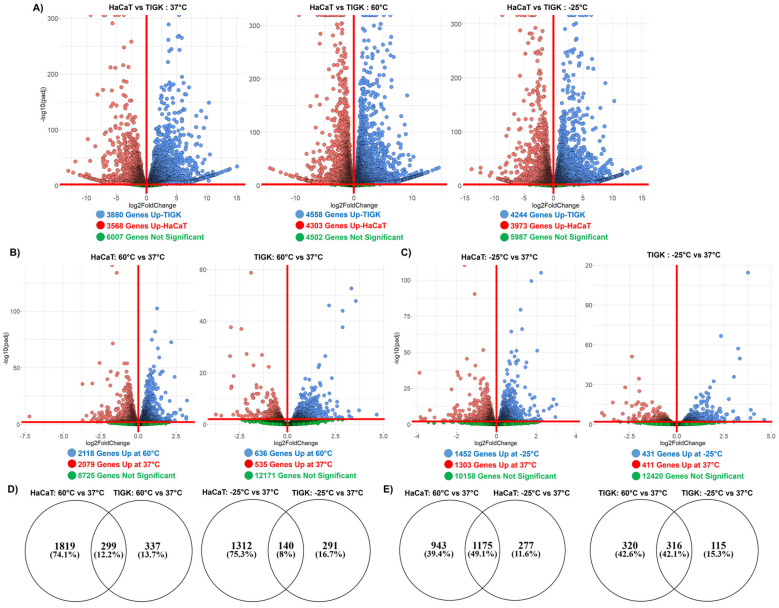
Oral and skin keratinocytes exhibit significant transcriptomic differences at baseline and following heat- or cold-induced injury. (**A**) Volcano plot showing the number of differentially expressed genes (DEGs) between HaCaTs and TIGKs at each temperature comparison: 37 °C, 60 °C, and −25 °C. (**B**) Volcano plot showing the number of DEGs in HaCaTs or TIGKs after exposure to 60 °C for 10 min relative to 37 °C or (**C**) to −25 °C for 10 min relative to 37 °C. (**D**) Venn diagram comparing the DEGs upregulated in HaCaTs and TIGKs after exposure to 60 °C for 10 min relative to 37 °C or to −25 °C for 10 min relative to 37 °C. (**E**) Venn diagram comparing the DEGs upregulated after exposure to 60 °C for 10 min relative to 37 °C and the DEGs upregulated after exposure to −25 °C for 10 min relative to 37 °C for HaCaTs and TIGKs.

The mRNA-sequencing data were examined to determine the number of DEGs (*p*.adj < 0.01, log (foldchange > 0 or <0) and the directionality of the differences between HaCaTs and TIGKs for the 3 conditions: 37 °C, 60 °C, and −25 °C. There were thousands of DEGs either up or downregulated between HaCaTs and TIGKs under these conditions (Figure 3A and Table 1). The lists of DEGs in HaCaTs and TIGKs at each temperature are listed in Supplementary Tables S1–S3. Genes with a positive log2FoldChange are upregulated in TIGKs, and genes with a negative log2FoldChange are upregulated in HaCaTs. We also performed Gene Ontology (GO) biological processes (BP) and molecular function (MF), and reactome pathway enrichment analysis on the DEGs of TIGKs and HaCaTs at 37 °C, 60 °C, and −25 °C (Supplementary Figures S1–S3). Enriched terms and their annotated genes are listed in Supplementary Tables S4–S21. Many of the enriched processes were related to cell differentiation, energy consumption, and responses to cell stress. Our preliminary analysis illustrates how distinct the transcriptome of HaCaTs and TIGKs are at baseline and after temperature-induced injury.

We next sought to identify the number of DEGs in response to temperature-induced injury in HaCaTs and TIGKs. Differential expression analysis was performed on the gene expression profiles of HaCaTs and TIGKs after exposure to 60 °C or −25 °C relative to their 37 °C baseline. The total number of DEGs (*p*.adj < 0.01, log (foldchange) > 0 or <0) for HaCaTs and TIGKs after exposure to 60 °C or −25 °C relative to their 37 °C baseline is shown in Figure 3B,C. There were thousands of DEGs either up or downregulated in HaCaTs exposed to heat or cold conditions compared to 37 °C (Figure 3B,C and Table 2) while there were hundreds of DEGs in TIGKs exposed to the same conditions (Figure 3B,C and Table 2). The lists of DEGs in HaCaTs and TIGKs following heat- or cold-induced injury relative to their baseline are listed in Supplementary Tables S22–S25. Downstream analyses and comparisons were performed only on genes that were upregulated following temperature-induced injury to identify potential processes or pathways activated in HaCaTs and TIGKs in response to heat and cold exposure.

We performed GO BP and MF and reactome pathway enrichment analysis on the differentially upregulated genes of TIGKs and HaCaTs at 60 °C or −25 °C relative to their own 37 °C (Supplementary Figures S4 and S5). Enriched terms and their annotated genes are listed in Supplementary Tables S26–S37. Heat-induced injury stimulated the expression of genes in HaCaTs annotated to processes related to cellular energy metabolism and antioxidant activity (Supplementary Figure S3A–C). Heat-induced injury stimulated the expression of genes in TIGKs annotated to processes related to cell differentiation and development (Supplementary Figure S3D–F). Cold-induced injury stimulated the expression of genes in HaCaTs related to cell differentiation and cellular responses to stress (Supplementary Figure S4A–C), while in TIGKs, genes related to cell differentiation and development and transcription were stimulated (Supplementary Figure S4D–F).

Our analysis of the DEGs following heat- or cold-induced injury suggests that HaCaTs exhibits a much more robust response to temperature-induced injury than TIGKs as they exhibit more DEGs (Figure 3B,C). To assess how similar the responses are for HaCaTs and TIGKs following temperature-induced injury, we compared the number of shared and unshared differentially upregulated genes in HaCaTs at 60 °C or −25 °C relative to 37 °C and in TIGKs at 60 °C or −25 °C relative to 37 °C (Figure 3D). For the response to heat-induced injury, there were only 299 shared differentially upregulated genes (12.2% of all differentially upregulated genes), while for the cold-induced injury, there were 140 shared differentially upregulated genes (8% of all differentially upregulated genes) (Figure 3D) between HaCaTs and TIGKs. While there are some similarities in the transcriptomic changes following injury in HaCaTs and TIGKs, a large proportion of genes are still differentially stimulated in a cell type-specific manner (Figure 3D).

Furthermore, we examined whether hot and cold-induced injuries elicited similar transcriptomic responses in HaCaTs and TIGKs. We compared the number of shared and unshared differentially upregulated genes in HaCaTs or TIGKs at 60 °C relative to 37 °C to HaCaTs at −25 °C relative to 37 °C (Figure 3E). In HaCaTs, there were 1175 genes differentially upregulated in both heat- or cold-induced injury (49.1% of all differentially upregulated genes) (Figure 3E). In TIGKs, there were 316 genes differentially upregulated in both heat- or cold-induced injury (42.1% of all differentially upregulated genes) (Figure 3E). These results suggest that heat- and cold-induced injuries may elicit similar transcriptomic responses in either HaCaTs or TIGKs.

### 2.4. HaCaTs and TIGKs Exhibit Differential Expression of Heat Shock Proteins

**Figure 4:**
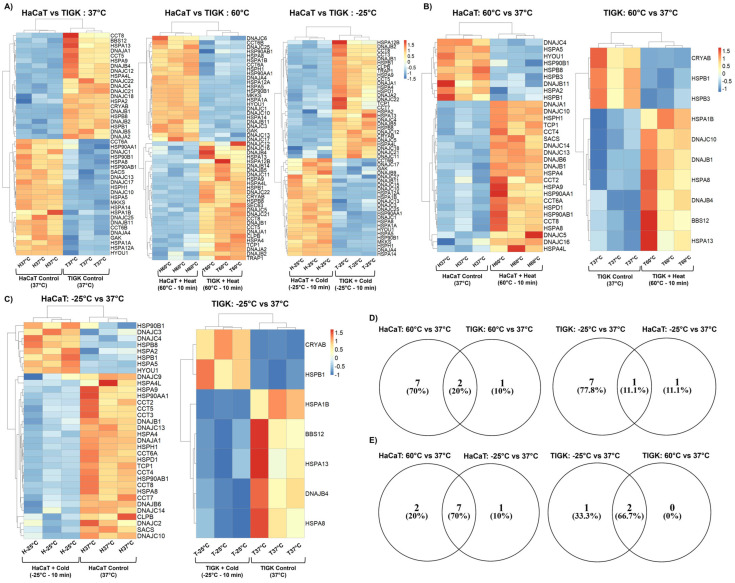
Oral and skin keratinocytes exhibit significant differences in their expression of heat shock proteins (HSPs) at baseline and following heat- or cold-induced injury. (**A**) Heatmap for all differentially expressed HSPs in HaCaTs and TIGKs at each temperature comparison: 37 °C, 60 °C, and −25 °C. (**B**) Heatmap of the HSPs differentially expressed in HaCaTs or TIGKs after exposure to 60 °C for 10 min relative to 37 °C or (**C**) to −25 °C for 10 min relative to 37 °C. Groups are denoted on both axes of the heatmap and each column or row represents an independent sample. (**D**) Venn diagram comparing the HSPs that are upregulated in HaCaTs and TIGKs after exposure to 60 °C for 10 min relative to 37 °C or to −25 °C for 10 min relative to 37 °C. (**E**) Venn diagram comparing the HSPs upregulated after exposure to 60 °C for 10 min relative to 37 °C and the HSPs upregulated after exposure to −25 °C for 10 min relative to 37 °C for HaCaTs and TIGKs.

HSPs are a well-conserved protein family that helps maintain cell homeostasis and protect cells from stresses, such as heat or cold exposure [5,20,23,24,25,26,27]. To determine if basal upregulation of specific HSPs in TIGKs may contribute to its enhanced tolerance to heat-induced injury, we identified 21, 27, and 29 upregulated HSPs in TIGKs relative to HaCaTs at 37 °C, 60 °C, and −25 °C respectively (Figure 4A). We also identified 23, 24, and 24 upregulated HSPs in HaCaTs relative to TIGKs at 37 °C, 60 °C, and −25 °C, respectively (Figure 4A and Table 3). The list of HSPs differentially expressed in HaCaTs and TIGKs at each temperature are listed in Supplementary Tables S38–S40. HSPs with a positive log2FoldChange are upregulated in TIGKs and HSPs with a negative log2FoldChange are upregulated in HaCaTs. GO BP and MF and reactome pathway enrichment analysis on the differentially expressed HSPs between TIGKs and HaCaTs at these temperatures are shown in Supplementary Figures S6–S8. Enriched terms and their annotated genes are listed in Supplementary Tables S41–S58. Many of the enriched processes were unsurprisingly related to responses to cellular stress and regulation of processes related to cell death (Supplementary Figures S6–S8).

We next sought to identify the number of HSPs differentially expressed in response to temperature-induced injury in HaCaTs and TIGKs. HaCaTs had 9 HSPs differentially upregulated and 22 HSPs differentially downregulated at 60 °C relative to 37 °C, while TIGKs had only 3 HSPs differentially upregulated and 7 HSPs differentially downregulated (Figure 4B and Table 4). HaCaTs had 8 HSPs differentially upregulated and 26 HSPs differentially downregulated at −25 °C relative to 37 °C, while TIGKs had only 2 HSPs differentially upregulated and 5 HSPs differentially downregulated (Figure 4C and Table 4). The lists of HSPs differentially expressed in HaCaTs and TIGKs following heat- or cold-induced injury relative to their baseline are listed in Supplementary Tables S59–S62. HSPs with a positive log2FoldChange are upregulated in HaCaTs or TIGKs at 60 °C or −25 °C relative to 37 °C and HSPs with a negative log2FoldChange are downregulated in HaCaTs or TIGKs at 60 °C or −25 °C relative to 37°.

Since elevated expression of HSPs is associated with the cellular response to heat and cold exposure [4,5,20,23,24,26,27,28,29,30,31], we focused further analyses on the HSPs that were upregulated following thermal and cold-induced injury. We performed GO BP and MF and reactome pathway enrichment analysis on the differentially upregulated HSPs of TIGKs and HaCaTs at 60 °C or −25 °C relative to their own 37 °C. These terms are shown in Supplementary Figures S9 and S10. Enriched terms and their annotated genes are listed in Supplementary Tables S63–S74. HSPs upregulated by either heat- or cold-induced injury in both HaCaTs and TIGKs annotated to processes related to cellular responses to stress or stimuli and regulation of cell death (Supplementary Figures S9 and S10).

We also compared the number of shared and unshared HSPs differentially upregulated in HaCaTs and TIGKs following heat- or cold-induced injury (Figure 4D). Of the HSPs stimulated in TIGKs following heat- or cold-induced injury, quite a few were also stimulated in HaCaTs (Figure 4D). For the 3 HSPs upregulated in TIGKs following heat-induced injury, 2 out of the 3 were also found to be upregulated in HaCaTs following heat-induced injury (20% of all differentially upregulated HSPs between HaCaTs and TIGKs) (Figure 4D). For the 2 HSPs upregulated in TIGKs following cold-induced injury, 1 out of the 2 were also found to be upregulated in HaCaTs following cold-induced injury (11.1% of all differentially upregulated HSPs between HaCaTs and TIGKs) (Figure 4D). Together, these results suggest that the response of TIGKs to heat- or cold-induced injuries has some similarities to HaCaTs in regards to the upregulation of HSPs. Our work also shows that HaCaTs upregulate more HSPs in response to heat and cold exposure than TIGKs. When comparing the HSPs differentially upregulated in either HaCaTs or TIGKs following heat- and cold-induced injury, we found that the majority of the HSPs activated in heat-induced injury and cold-induced injury were shared for both cell types (Figure 4E). This further suggests that both heat- and cold-induced injuries might illicit similar transcriptomic responses.

### 2.5. Constitutively Expressed Heat Shock Proteins in Uninjured TIGKs May Enhance Their Resistance to Temperature Injury

**Figure 5:**
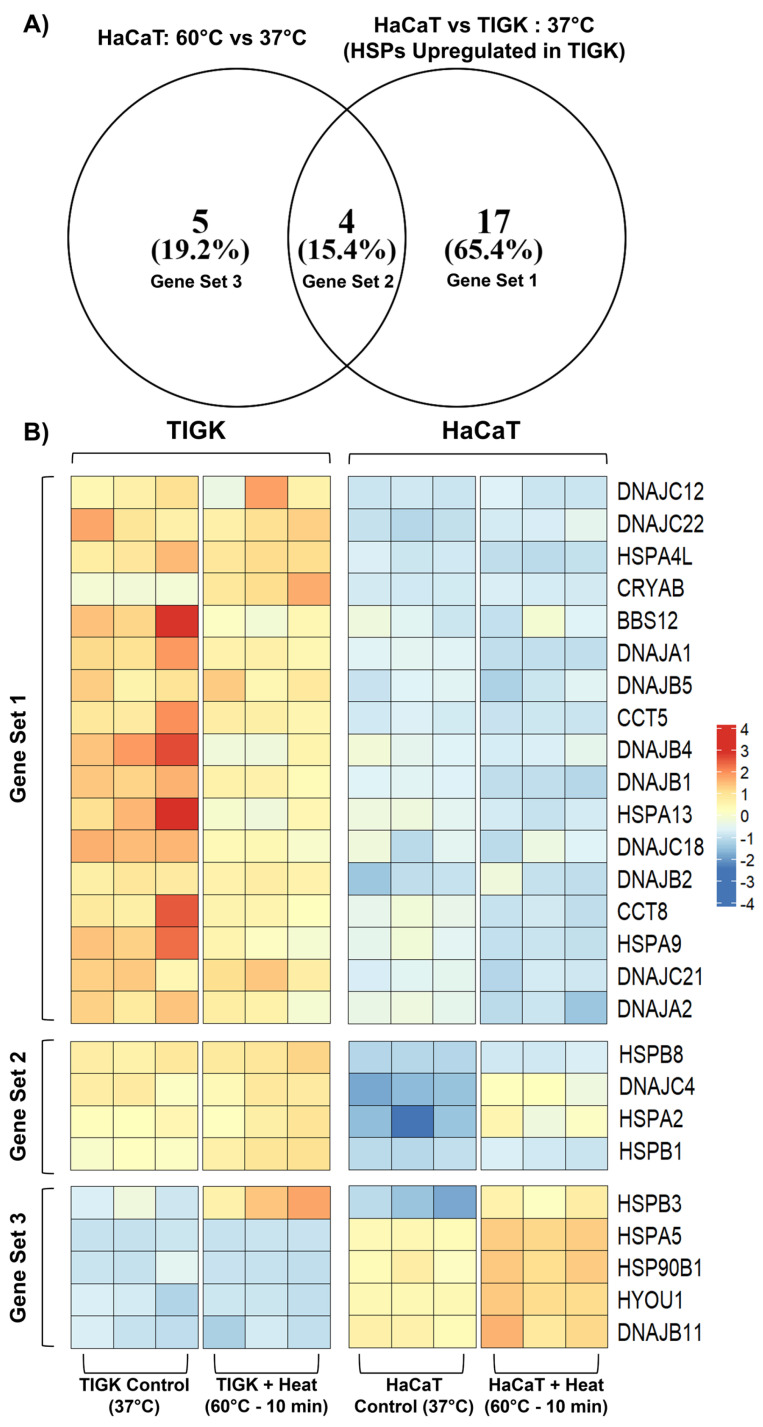
Oral keratinocytes have an advantageous expression profile of heat shock proteins that relate to survival at baseline. (**A**) Venn diagram comparing the shared HSPs that are upregulated in TIGKs relative to HaCaTs at 37 °C and in HaCaTs after exposure to 60 °C for 10 min relative to 37 °C. (**B**) Heatmap showing expression of HSPs upregulated in TIGKs relative to HaCaTs at 37 °C and in HaCaTs after exposure to 60 °C for 10 min relative to 37 °C. Groups are denoted on both axes of the heatmap and each column or row represents an independent sample.

To examine why TIGKs might have an enhanced heat tolerance over HaCaTs, despite having far fewer HSPs differentially upregulated after heat-induced injury, we conducted a comparative analysis between the HSPs upregulated in TIGKs relative to HaCaTs at 37 °C to the HSPs differentially upregulated in HaCaTs following heat-induced injury. This analysis led to the identification of 4 HSPs (HSPB8, DNAJC4, HSPA2, and HSPB1) that were basally upregulated in TIGKs relative to HaCaTs at 37 °C and differentially expressed in HaCaTs at 60 °C relative to 37 °C (Gene Set 2) (Figure 5). The Gene Sets associated with this comparison are listed in Table 5. We also identified 4 HSPs (HSPB8, DNAJC4, HSPA2, and HSPB1) that were basally upregulated in TIGKs relative to HaCaTs at 37 °C and differentially expressed in HaCaTs at −25 °C relative to 37 °C (Gene Set 5) (Supplementary Figure S11). The Gene Sets associated with this comparison are listed in Supplementary Table S75. Gene Set 2 comprises about half of the HSPs that become differentially activated in HaCaTs following heat-induced injury (Figure 5). It is possible that Gene Set 2 may help confer the enhanced heat tolerance of TIGKs at baseline and may not require upregulation following heat-induced injury. Gene Set 2 and Gene Set 5 consisted of the same HSPs, suggesting that they are involved in the cellular responses to both heat- and cold-induced injury in HaCaTs and TIGKs.

## 3. Discussion

Although skin and oral mucosal wounds proceed through similar stages of healing in vivo, oral mucosal wounds have been shown to heal more rapidly with faster re-epithelialization and less scarring and inflammation [6,7,10,11,12,13]. Another form of injury experienced by the skin and oral mucosa is caused by extreme heat and cold exposure, such as burns or frostbite [3,32,33,34,35]. Some studies have demonstrated that skin keratinocyte exposure to heat and cold induces the synthesis of stress proteins, such as HSPs, and may result in cell death [4,5,26,27]. To our knowledge, there are no studies assessing oral keratinocyte response to heat and cold exposure nor are there studies comparing the response of skin and oral keratinocytes to temperature-induced injury [3,4,5,20,26,27,36,37]. Our assays assess the temperature tolerance of skin and oral keratinocytes. Furthermore, our mRNA-sequencing analysis offers a global view of gene expression in skin and oral keratinocytes as well as a comprehensive assessment of the transcriptomic differences that underlie their differential responses to temperature-induced injuries.

HaCaTs and TIGKs did not exhibit any discernible differences in their ability to survive cold conditions. TIGKs did, however, have a significantly higher viability following heat exposure, suggesting a higher degree of heat tolerance as compared to HaCaTs. TIGKs did not have more HSPs differentially upregulated following heat-induced injury as compared to HaCaTs nor were there more HSPs differentially expressed in TIGKs relative to HaCaTs at 37 °C or 60 °C. Perhaps, TIGKs might only require the activation of a select few HSPs following temperature-induced cell stress or a greater degree of cell stress is needed to induce the activation of more HSPs. Another possibility is that at baseline, TIGKs exhibit an expression profile of HSPs that allows it to have an enhanced heat tolerance. Comparative analysis between the HSPs basally upregulated in TIGKs relative to HaCaTs found that some of these HSPs also get activated in HaCaTs after temperature-induced injury. These HSPs are HSPB8, DNAJC4, HSPA2, and HSPB1. Studies in cancers suggest that these HSPs are primarily involved in cell survival, differentiation potential, and prevention of apoptosis [20,28,36,38,39,40,41], which are indispensable programs for preventing cell death following cell stress. This expression profile of HSPs in TIGKs may therefore confer its enhanced thermal tolerance over HaCaTs and prepare them to better respond to temperature-induced injury, even without an upregulation in their expression following heat exposure. Outside of temperature-induced injuries, oral keratinocytes may also be better equipped to respond to other forms of injury as well. Future analyses comparing the response of oral and skin keratinocytes to different types of injury, such as physical trauma, oxidative damage, and UV radiation are warranted. These analyses would provide further insight into the intrinsic differences between the two cell types and how they may influence their respective tissue healing outcomes.

Analysis of the DEGs between HaCaTs and TIGKs at baseline and following heat- or cold-inducing injury conditions suggests that their transcriptomic responses to temperature-induced injuries may be distinct. Many of the genes differentially upregulated following heat or cold injury are unshared between HaCaTs and TIGKs. Despite these differences, these genes were enriched for similar processes, such as cell differentiation, energy consumption, and cellular responses to stress. Many of these processes are greatly affected in other cell types following heat- or cold-induced injury [29,42,43,44,45]. These transcriptomic changes are likely a compensatory mechanism that helps keratinocytes adapt to fluctuations in temperatures to sustain epithelium viability [3,5,23,26]. However, under prolonged or more severe temperature exposures, significant cell death still occurs as the compensation cannot overcome the cell stress. When comparing the transcriptomic response that heat and cold exposure elicited, we noticed that many of the DEGs and HSPs differentially upregulated following heat exposure were similar to those upregulated following cold exposure for both cell types. This suggests that in addition to specific responses to heat or cold exposure, a common or conserved response to temperature change, or perhaps any stimuli that might cause cell stress, exists.

The lack of significant differences between HaCaTs and TIGKs in their cold tolerance could be due to the mechanism by which cold injuries occur in cells. As cells freeze, ice crystals form both intra- and extracellularly, leading to damage to intracellular structures and the cell membrane and, ultimately, cell death [46,47]. Although stress response proteins, such as HSPs, may be activated, once the cell is completely frozen, cell death usually follows [46,47]. This pathway would be similar for both HaCaTs and TIGKs in the longer cold exposure times in our assays. Additional studies utilizing different cold exposure conditions will be necessary to determine if differences in cold tolerance exist between HaCaTs and TIGKs.

It is important to note that our analyses are limited in scope by the temperatures chosen. Much of the transcriptomic changes reflect a response to temperatures that may ultimately result in cell death. More moderate temperatures could elicit different transcriptomic responses and may even be beneficial for keratinocytes, as studies in other cell types have already shown that mild heat exposure can promote proliferation and differentiation [48,49,50]. However, our focus was to analyze temperatures that would certainly lead to burn or freezing injuries and the response of epidermal cells. It is also important to note that although we focused on HSPs, other stress proteins including RNA chaperone proteins and proteins of the endoplasmic reticulum, may be activated following heat and cold exposure [51]. Additionally, non-stress proteins that were activated or inactivated may also play a role in cellular responses to stress. Analysis of these other genes may be important for determining the intrinsic differences between how oral and skin keratinocytes respond to cell stressors. Furthermore, we focused on upregulated genes and HSPs following temperature-induced injury based on the assumption that cellular responses to cell stress would be related to activation of adaptive processes and induction of HSPs [4,5,20,23,24,26,27,28,29,30,31]. We understand it is also important to consider both up and downregulated genes for a holistic understanding of the biological response of keratinocytes to heat and cold exposure. Future analysis of the downregulated genes is therefore warranted, as well as gene set enrichment analysis, which considers both up and downregulated genes when identifying significantly enriched pathways [52,53,54]. Our studies also compare isolated cells grown in vitro and on cell-culture-treated polystyrene, which may not be reflective of how they function in their native tissue or when other cell types are present. A future study utilizing single-cell RNA-sequencing of oral and skin tissue at baseline and after cold or heat exposure could be performed to validate our findings.

In our studies, we utilized immortalized keratinocyte cell lines HaCaTs and TIGKs, which have been commonly used to study keratinocyte behavior in skin and oral wound healing in vitro [55,56,57,58,59,60,61,62]. Using these immortalized keratinocyte cell lines helps to avoid the complications associated with using primary skin and oral keratinocytes in vitro, such as their short lifespan and sample variability [59,60,61]. Moreover, HaCaTs and TIGKs have been shown to exhibit similar morphogenesis, expression of cytokeratins, as well as other major surface markers, and functional characteristics, such as response to inflammatory cytokines and proliferative capacities, as their parental primary cell [59,60,61,63]. To assess how similar HaCaTs and TIGKs are to their parental primary cells at the transcriptomic levels, we compared the top 1000 genes for HaCaTs and TIGKs at baseline (37 °C) to the top 1000 genes for normal human epidermal keratinocytes (NHEK) (GSE185309) and normal human oral keratinocytes (NHOK) (GSE262505) [64,65], respectively. Many genes were shared between HaCaTs and NHEK or TIGKs and NHOK (Supplementary Figure S12A,B). Reactome pathway enrichment analysis of the top 1000 genes in HaCaTs, TIGKs, NHEK, and NHOK found that many terms were shared (Supplementary Tables S76–S79 and Supplementary Figure S12C–F). Lastly, genes previously shown to be more highly expressed in primary oral keratinocytes versus skin keratinocytes, such as PITX1, PITX2, SOX2, PAX9, SIM2, and IGFBP2, were also found to be higher in TIGKs versus HaCaTs (Supplementary Tables S1 and S80) [8,10,62,66]. Altogether, this implies that HaCaTs and TIGKs maintain similarity, at least in the transcriptome, to their parental primary cell.

Still, the level of similarity of the immortalized HaCaTs and TIGKs responses to temperature-induced injury to that of their parental primary cell needs more investigation. It was previously shown that chronic exposure to heat (40 °C) may have a tumorigenic effect on HaCaTs [67], which has not been observed in primary skin keratinocytes. To our knowledge, no such findings have been observed in TIGKs or primary oral keratinocytes. Our studies assessed viability and cell death following much higher temperatures for a shorter time, possibly circumventing the aforementioned tumorigenic mechanisms. Another limitation of our study is that TIGKs and HaCaTs underwent different immortalization processes, with TIGKs being telomerase-immortalized and HaCaTs being spontaneously immortalized [59,63]. There may also still be underlying genetic and epigenetic properties influencing their responses to temperature-induced injury [59,63,68]. While these factors may have a confounding effect on our results and thereby limit the scope of our findings, we believe that comparing HaCaTs and TIGKs still provides a valuable starting point for understanding the differences between oral and skin keratinocytes. Future studies that compare primary skin and oral keratinocytes are needed to validate the use of HaCaTs and TIGKs as a model system.

Overall, our studies demonstrate that oral and skin keratinocytes have distinct responses to temperature-induced injury and that the differential expression of HSPs may be one factor in mediating their resistance to cellular stresses. Although skin keratinocytes exhibited a more robust transcriptomic response to injury, oral keratinocytes had an enhanced resistance to heat-induced injury. Our analysis suggests this may be due to oral keratinocytes having a more advantageous gene expression profile of HSPs at baseline. A more detailed examination of these HSPs as well as other stress-related proteins is warranted to infer their role in adapting to cellular stresses. Lastly, our analyses show how responsive keratinocytes are to heat and cold, which may contribute to the ability of the oral mucosa and skin epidermis to adapt to temperature changes [3,69,70,71].

## 4. Materials and Methods

### 4.1. Skin and Oral Keratinocyte Culture

Spontaneously immortalized skin keratinocytes (HaCaTs) were purchased from AddexBio (San Diego, CA, USA). Dr. Richard J. Lamont at the University of Louisville kindly provided the human telomerase-immortalized gingival keratinocyte (TIGKs) cell line under a material transfer agreement. Both cell lines were cultured in DermaLife K Keratinocyte Complete Medium (Lifeline Cell Technology, Frederick, MD, USA), which contained D-glucose (6 mM), insulin (5 µg/mL), L-glutamine (6 mM), epinephrine (1 µM), apo-transferrin (5 µg/mL), TGF-α (0.5 ng/mL), pituitary extract (0.4%), and hydrocortisone hemisuccinate (100 ng/mL). Cells were cultured in an incubator at 37 °C with 5% CO_2_.

### 4.2. Skin and Oral Keratinocyte Temperature-Induced Injury and Viability

HaCaTs and TIGKs were seeded into a 6-well plate. Once cells reached confluence, 2 mL of 60 °C pre-heated or ice-cold culture medium was added to each well. The plates were then subjected to heat- or cold-inducing injury conditions. Heat-inducing injury conditions were simulated by placing plates in a 60 °C water bath for 10 or 20 min. Cold-inducing injury conditions were simulated by placing plates in a −25 °C freezer for 10, 20, or 60 min. Digital images of the cells were captured. Trypan blue was added to the single-cell suspension disassociated from the plate using TrypLE (Thermo Fisher Scientific, Waltham, MA, USA), and viability was recorded using a TC20 Automated Cell Counter (Bio-Rad, Hercules, CA, USA). Cell viability was immediately assessed following heat- and cold-induced injury. Cells cultured at 37 °C served as a general control, and cells cultured under frozen conditions in a medium with 10% dimethyl sulfoxide (DMSO) served as an additional control. Each condition was tested with 3 replicates, and the experiment was repeated 2–4 times.

### 4.3. Assessment of Cellular Apoptosis

Following the temperature-induced injury to HaCaTs and TIGKs, cell death including apoptosis and necrosis was assessed by using an FITC Annexin V Apoptosis Detection Kit (BD Bioscience, San Jose, CA, USA). Briefly, harvested cells were washed with binding buffer, re-suspended in 100 μL of binding buffer, and incubated with FITC-conjugated Annexin V for 15 min at room temperature. After washing, 5 μL of propidium iodide (PI) was added. The cells were then immediately subjected to flow cytometry analysis using LSR Fortessa (BD Bioscience). Results were analyzed using FlowJo™ v10.10 Software (BD Life Sciences, Franklin Lakes, NJ, USA). Cell debris and doublets were gated out in the analysis. Each condition was tested with 3 replicates and the experiment was repeated 1 time.

### 4.4. RNA Extraction, Library Preparation, and Data Processing for mRNA-Sequencing

We performed mRNA-sequencing analysis to molecularly characterize the differences in response between HaCaTs and TIGKs to heat or cold injury. RNA was collected from HaCaTs and TIGKs immediately after being exposed to 60 °C or −25 °C for 10 min to ensure ample time for a transcriptomic response to each temperature yet to maintain enough viable cells with good RNA integrity for mRNA-sequencing. RNA from HaCaTs and TIGKs cultured in normal 37 °C conditions were used as baseline. There were 3 biological replicates for each cell type for each condition. Total RNA was isolated using TRIzol (Invitrogen, Waltham, MA, USA), purified with an RNA Clean and Concentrator-25 kit (Zymo, Tustin, CA, USA), and treated with DNAse (ThermoFisher Scientific, Waltham, MA, USA). RNA integrity numbers (RIN) were checked with Agilent Technologies 2100 Bioanalyzer (Agilent Technologies, Santa Clara, CA, USA). All samples had an RIN between 8.9–9.4.

Libraries generated for mRNA-sequencing were checked with Qubit and real-time PCR for quantification and bioanalyzer for size distribution detection. Quantified libraries were pooled and sequenced on Illumina sequencing platforms (Illumina, Omaha, NE, USA) and clean data were obtained by using in-house Perl scripts from Novogene (Novogene America, Sacramento, CA, USA). Hisat2 (v2.0.5) was used to index and map reads to the human reference genome (GRCh38/hg38), and the mapped reads were assembled by StringTie (v1.3.3b) [72]. Read numbers mapped to each gene were counted in R using the FeatureCounts package (v1.5.0-p3) and an FPKM of each gene was calculated [73]. The library preparation, genome mapping, and raw gene counts for mRNA-sequencing analysis were performed by Novogene (Novogene America, Sacramento, CA, USA).

All analyses described below were performed by us using R (v4.3.2) with Bioconductor v3.17. Differential expression analysis of the raw count data generated by Novogene (Novogene America) was performed by utilizing the DESeq2 package (v1.20.0) in R [74]. Only genes with at least an average raw count of 10 across all samples were analyzed. The resulting *p*-values were adjusted using the Benjamini and Hochberg’s approach for controlling the false discovery rate (FDR) [74,75]. Genes with an adjusted *p*-value (*p*.adj) < 0.05 were assigned as being a DEG after DESeq2 analysis. Normalized count values for the DEGs in each sample were generated for each comparison.

For data visualization and principal component analysis (PCA), count data underwent variance stabilized transformation using the vst() function in DESeq2 and was saved. PCA was then plotted using each sample’s top 500 highly variable genes. A Pearson’s correlation coefficient based on the computed Euclidean distance matrix and hierarchical clustering using the complete agglomeration method was also performed. Pearson’s correlation coefficients between samples of the same experimental group were averaged to form an aggregate value and then visualized as a heatmap. We then assessed differentially regulated genes in HaCaTs and TIGKs at baseline (37 °C) and after either heat- or cold-induced injury using the following parameters: *p*.adj < 0.01 and log (foldchange) > 0 or log (foldchange) < 0. Their responses to temperature-induced injury were compared by identifying the DEGs in HaCaTs or TIGKs after 10 min under 60 °C or −25 °C conditions relative to their own baseline (37 °C) using the following parameters: *p*.adj < 0.01 and log (foldchange) > 0 or log (foldchange) < 0. The list of DEGs with our parameters were uploaded to GSE289231. Differentially expressed HSPs between HaCaTs and TIGKs at baseline (37 °C), following heat- or cold-induced injury (60 °C and −25 °C), and in response to heat or cold injury (60 °C or −25 °C vs. 37 °C) were identified by extracting out gene names corresponding to HUGO Gene Nomenclature Committee’s (HGNC’s) established gene nomenclature for heat shock proteins [76,77,78]. Z-scores for genes were generated using normalized gene counts and plotted via pheatmap(), version 1.0.12. [79]. Venn diagrams were made with Venny [80].

### 4.5. Comparison of HaCaTs and TIGKs to Primary Skin and Oral Keratinocytes

Raw count matrices from mRNA-sequencing of NHEK were acquired from GSE185309 [64], while raw count matrices from mRNA-sequencing of NHOK were acquired from a separate study, GSE262505 [65]. Raw counts for each gene identified in HaCaTs and TIGKs at baseline (37 °C) from our generated mRNA-sequencing dataset and in the samples from GSE185309 and GSE262505, were averaged, and only genes with an average count > 10 across all samples in each study were kept for further analysis. The top 1000 genes based on average counts were identified in HaCaTs, TIGKs, NHEK (GSE185309), and NHOK (GSE262505). Reactome pathway enrichment analysis was performed on these 1000 genes to determine their biological significance. Additionally, we examined how many of the top 1000 genes were shared or different between the following groups: (1) HaCaTs and NHEK (GSE185309) and (2) TIGKs and NHOK (GSE262505).

### 4.6. Gene Ontology and Reactome Pathway Enrichment Analysis of DEGs

GO BP, MF, and reactome pathway enrichment analysis were performed with EnrichR [81,82,83]. *p*-values were adjusted using the Benjamini and Hochberg’s approach for controlling the FDR. BP or MF GO and reactome terms with *p*.adj < 0.05 were considered significant.

### 4.7. Statistical Analysis

Data are expressed as mean ± standard deviation, and their normality was evaluated using the Shapiro–Wilk tests. Statistical comparisons were performed using a two-way ANOVA or multiple two-tailed unpaired *t*-test with Welch’s correction followed by a two-stage linear step-up procedure of Benjamini, Krieger, and Yekutieli post-hoc testing using GraphPad Prism version 8.0 (GraphPad, San Diego, CA, USA). Outliers were removed by Grub’s test with an alpha significance level of 0.05. *p*-values less than 0.05 were considered statistically significant.

## Figures and Tables

**Table 1:** Differentially expressed genes between HaCaTs and TIGKs at baseline (37 °C) and after temperature-induced injury (60 °C or −25 °C).

	Control (37 °C)	Heat-Induced Injury (60 °C)	Cold-Induced Injury (−25 °C)
Upregulated in HaCaTs	3568	4303	3973
Upregulated in TIGKs	3880	4558	4244
Not Significant	6007	4502	5098

**Table 2:** Differentially expressed genes between HaCaTs and TIGKs after temperature-induced injury (60 °C or −25 °C) relative to their own baseline (37 °C).

	HaCaTs		TIGKs	
	Heat-Induced Injury Response (60 °C vs. 37 °C)	Cold-Induced Injury Response (−25 °C vs. 37 °C)	Heat-Induced Injury Response (60 °C vs. 37 °C)	Cold-Induced Injury Response (−25 °C vs. 37 °C)
Upregulated	2118	1452	636	431
Downregulated	2079	1303	535	411
Not Significant	8725	10,158	12,171	12,420

**Table 3:** Differentially expressed heat shock proteins between HaCaTs and TIGKs at baseline (37°C) and after temperature-induced injury (60 °C or −25 °C).

	Control (37 °C)	Heat-Induced Injury (60 °C)	Cold-Induced Injury (−25 °C)
Upregulated in HaCaTs	23	24	29
Upregulated in TIGKs	21	27	24

**Table 4:** Differentially expressed heat shock proteins in HaCaTs or TIGKs after temperature-induced (60 °C or −25 °C) injury relative to their own baseline (37 °C).

	HaCaTs		TIGKs	
	Heat-Induced Injury Response (60 °C vs. 37 °C)	Cold-Induced Injury Response (−25 °C vs. 37 °C)	Heat-Induced Injury Response (60 °C vs. 37 °C)	Cold-Induced Injury Response (−25 °C vs. 37 °C)
Upregulated	9	8	3	2
Downregulated	22	26	7	5

**Table 5:** Differentially expressed heat shock proteins (HSPs) for each gene set.

Gene Set	Gene Set Meaning	HSPs
1	HSPs activated in HaCaTs at −25 °C relative to 37 °C	*DNAJC12*, *DNAJC22*, *HSPA4L*, *CRYAB*, *BBS12*, *DNAJA1*, *DNAJB5*, *CCT5*, *DNAJB4*, *DNAJB1*, *HSPA13*, *DNAJC18*, *DNAJB2*, *CCT8*, *HSPA9*, *DNAJC21*, *DNAJA2*
2	HSPs activated in HaCaTs at −25 °C relative to 37 °C and differentially upregulated in TIGKs relative to HaCaTs at 37 °C	*HSPB8*, *DNAJC4*, *HSPA2*, *HSPB1*
3	HSPs differentially upregulated in TIGKs relative to HaCaTs at 37 °C	*HSPB3*, *HSPA5*, *HSP90B1*, *HYOU1*, *DNAJB11*

## Data Availability

All sequencing-related data, including raw counts and our DESeq2 differential expression analysis, are available in NCBI GEO with an accession number GSE289231. The results of the analyses performed on our mRNA-sequencing data stated in this manuscript may be found in our supplementary materials. The R code used in this manuscript is available on github: https://github.com/ChenHanMDPhD/Temperature-Induced-Injury-to-HaCaT-and-TIGK/blob/main/Thermal%20Injury%20Code_FINAL.R, accessed on 19 March 2025. The mRNA-sequencing data utilized from GSE185309 and GSE262505 are available at: https://www.ncbi.nlm.nih.gov/geo/query/acc.cgi?acc=GSE185309, accessed on 16 March 2025 and https://www.ncbi.nlm.nih.gov/geo/query/acc.cgi?acc=GSE262505, accessed on 16 March 2025.

## Supplementary Materials

The following supporting information can be downloaded at: https://www.mdpi.com/article/10.3390/ijms26072851/s1.
