# Supplementary material for: Differential Temperature-Induced Responses in Immortalized Oral and Skin Keratinocytes
Source: Int J Mol Sci. 2025 Mar 21;26(7):2851. doi: 10.3390/ijms26072851 (PMC11988828; doi:10.3390/ijms26072851)
Supplement: Supplementary file 1 [file ijms-26-02851-s001.zip › Supplementary Figures_03142025.pptx]

## Slide 1
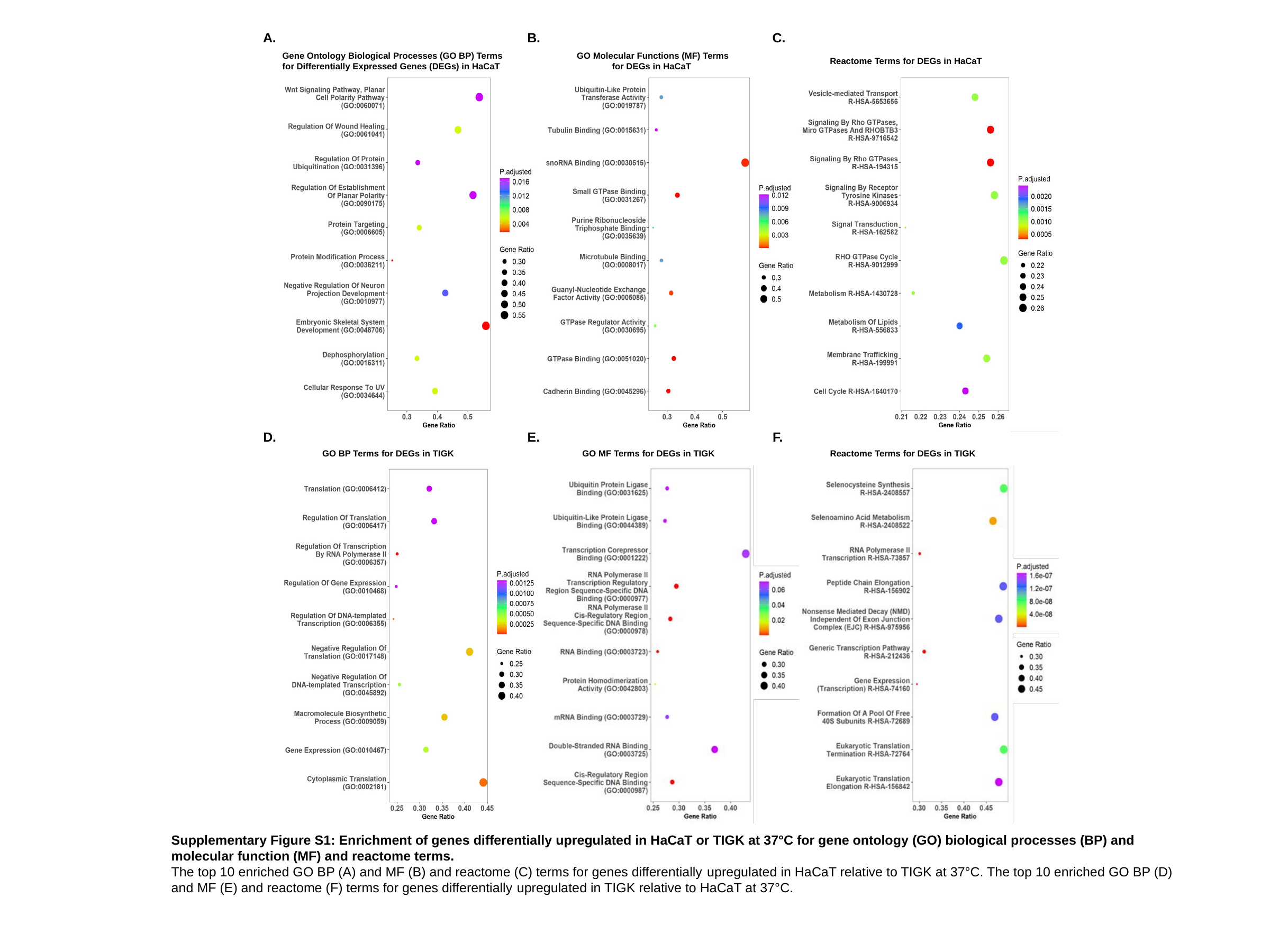

C.
A.
B.
Gene Ontology Biological Processes (GO BP) Terms for Differentially Expressed Genes (DEGs) in HaCaT
GO Molecular Functions (MF) Terms for DEGs in HaCaT
Reactome Terms for DEGs in HaCaT
F.
D.
E.
GO BP Terms for DEGs in TIGK
GO MF Terms for DEGs in TIGK
Reactome Terms for DEGs in TIGK
Supplementary Figure S1: Enrichment of genes differentially upregulated in HaCaT or TIGK at 37°C for gene ontology (GO) biological processes (BP) and molecular function (MF) and reactome terms.
The top 10 enriched GO BP (A) and MF (B) and reactome (C) terms for genes differentially upregulated in HaCaT relative to TIGK at 37°C. The top 10 enriched GO BP (D) and MF (E) and reactome (F) terms for genes differentially upregulated in TIGK relative to HaCaT at 37°C.

## Slide 2
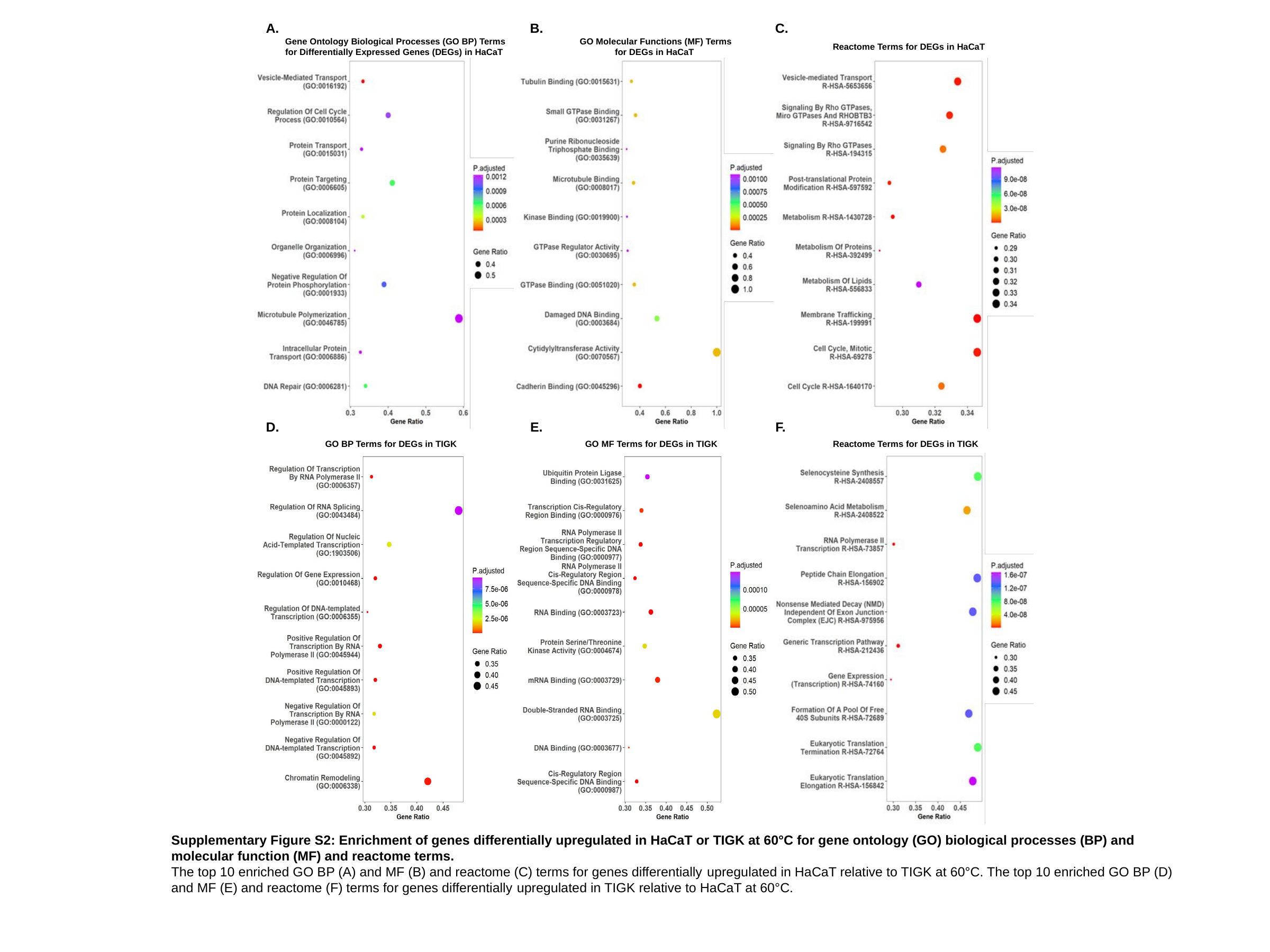

C.
A.
B.
Gene Ontology Biological Processes (GO BP) Terms for Differentially Expressed Genes (DEGs) in HaCaT
GO Molecular Functions (MF) Terms for DEGs in HaCaT
Reactome Terms for DEGs in HaCaT
F.
D.
E.
GO BP Terms for DEGs in TIGK
GO MF Terms for DEGs in TIGK
Reactome Terms for DEGs in TIGK
Supplementary Figure S2: Enrichment of genes differentially upregulated in HaCaT or TIGK at 60°C for gene ontology (GO) biological processes (BP) and molecular function (MF) and reactome terms.
The top 10 enriched GO BP (A) and MF (B) and reactome (C) terms for genes differentially upregulated in HaCaT relative to TIGK at 60°C. The top 10 enriched GO BP (D) and MF (E) and reactome (F) terms for genes differentially upregulated in TIGK relative to HaCaT at 60°C.

## Slide 3
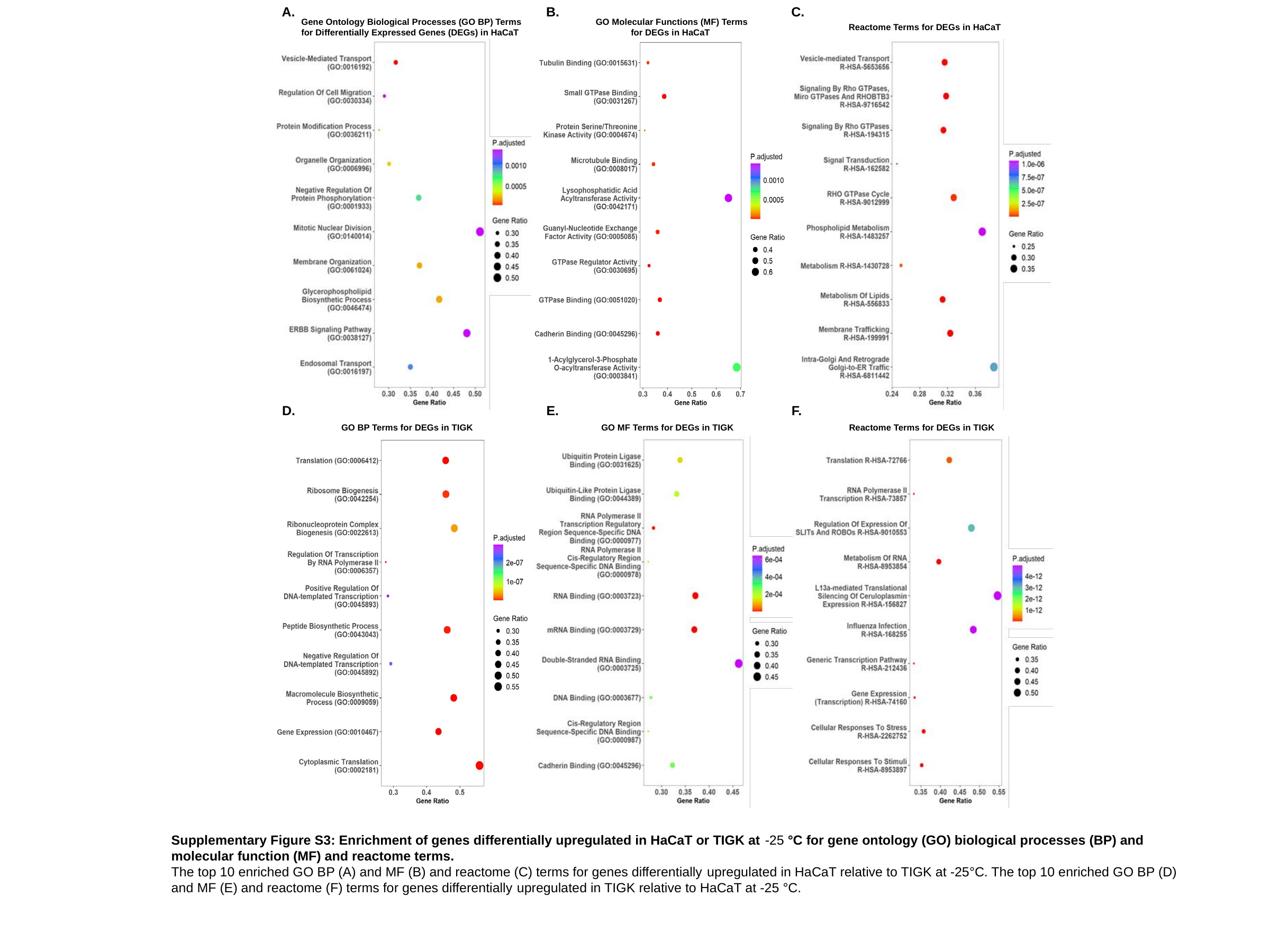

C.
A.
B.
Gene Ontology Biological Processes (GO BP) Terms for Differentially Expressed Genes (DEGs) in HaCaT
GO Molecular Functions (MF) Terms for DEGs in HaCaT
Reactome Terms for DEGs in HaCaT
F.
D.
E.
GO BP Terms for DEGs in TIGK
GO MF Terms for DEGs in TIGK
Reactome Terms for DEGs in TIGK
Supplementary Figure S3: Enrichment of genes differentially upregulated in HaCaT or TIGK at -25 °C for gene ontology (GO) biological processes (BP) and molecular function (MF) and reactome terms.
The top 10 enriched GO BP (A) and MF (B) and reactome (C) terms for genes differentially upregulated in HaCaT relative to TIGK at -25°C. The top 10 enriched GO BP (D) and MF (E) and reactome (F) terms for genes differentially upregulated in TIGK relative to HaCaT at -25 °C.

## Slide 4
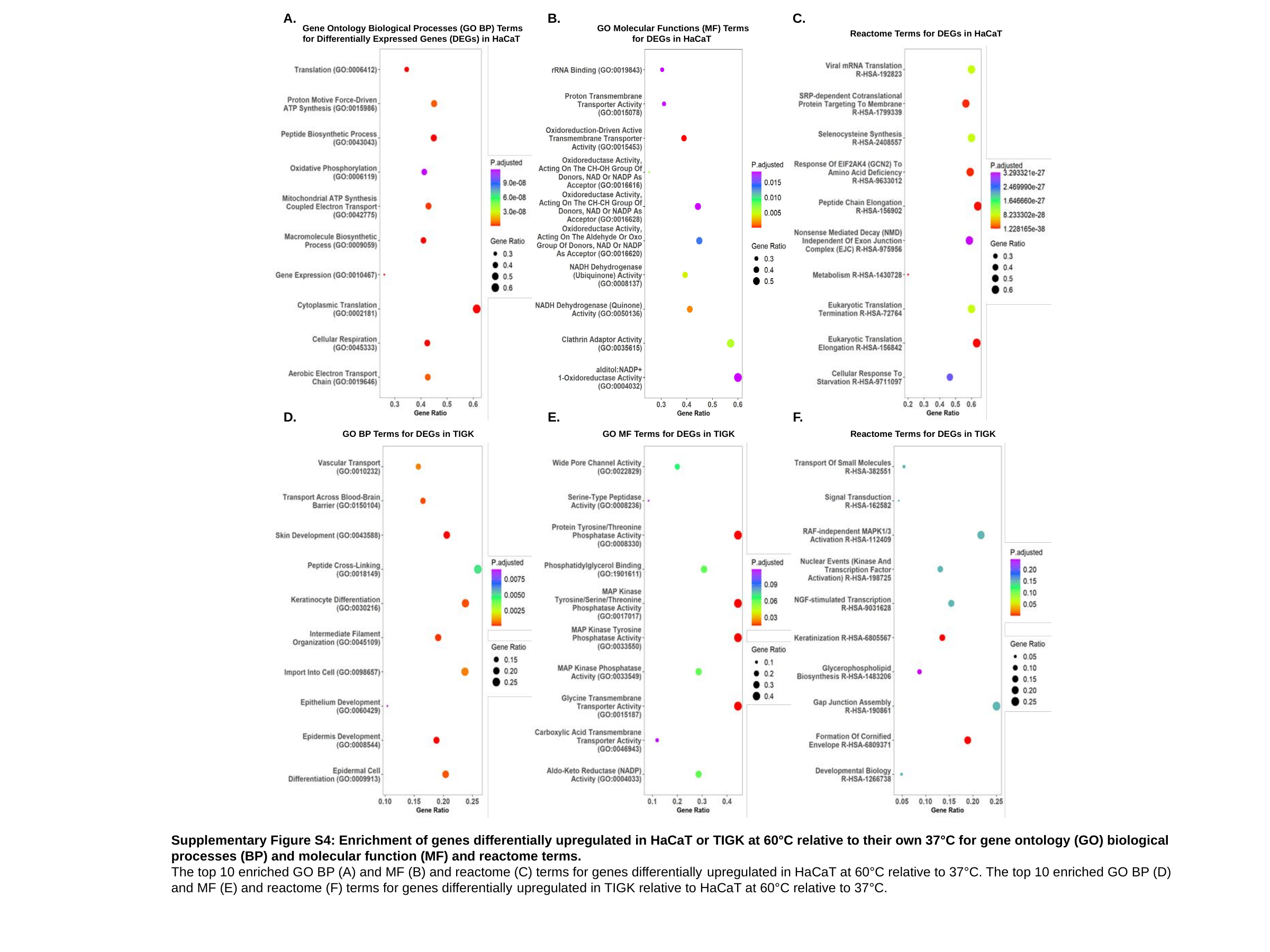

C.
A.
B.
Gene Ontology Biological Processes (GO BP) Terms for Differentially Expressed Genes (DEGs) in HaCaT
GO Molecular Functions (MF) Terms for DEGs in HaCaT
Reactome Terms for DEGs in HaCaT
F.
D.
E.
GO BP Terms for DEGs in TIGK
GO MF Terms for DEGs in TIGK
Reactome Terms for DEGs in TIGK
Supplementary Figure S4: Enrichment of genes differentially upregulated in HaCaT or TIGK at 60°C relative to their own 37°C for gene ontology (GO) biological processes (BP) and molecular function (MF) and reactome terms.
The top 10 enriched GO BP (A) and MF (B) and reactome (C) terms for genes differentially upregulated in HaCaT at 60°C relative to 37°C. The top 10 enriched GO BP (D) and MF (E) and reactome (F) terms for genes differentially upregulated in TIGK relative to HaCaT at 60°C relative to 37°C.

## Slide 5
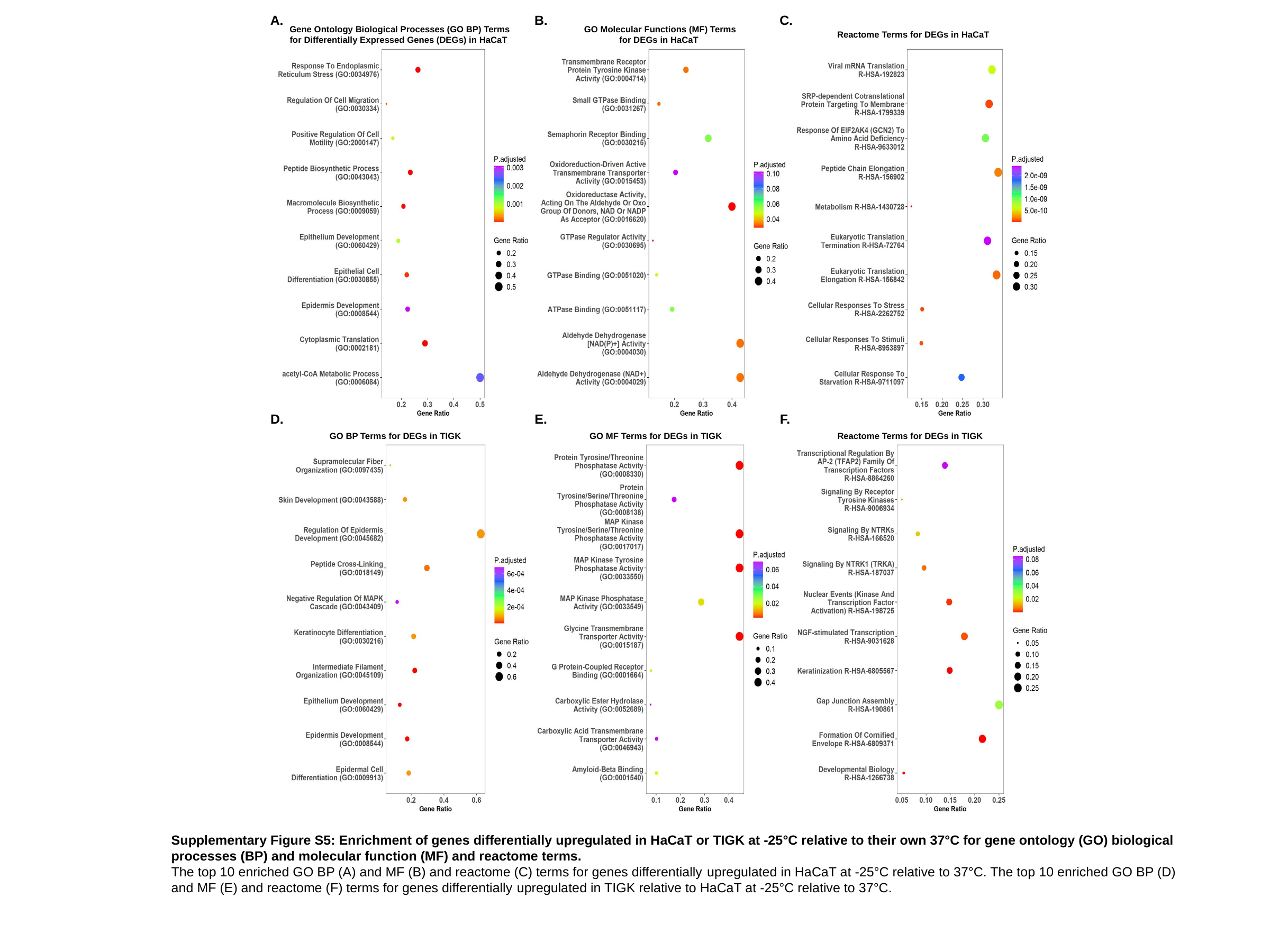

C.
A.
B.
Gene Ontology Biological Processes (GO BP) Terms for Differentially Expressed Genes (DEGs) in HaCaT
GO Molecular Functions (MF) Terms for DEGs in HaCaT
Reactome Terms for DEGs in HaCaT
F.
D.
E.
GO BP Terms for DEGs in TIGK
GO MF Terms for DEGs in TIGK
Reactome Terms for DEGs in TIGK
Supplementary Figure S5: Enrichment of genes differentially upregulated in HaCaT or TIGK at -25°C relative to their own 37°C for gene ontology (GO) biological processes (BP) and molecular function (MF) and reactome terms.
The top 10 enriched GO BP (A) and MF (B) and reactome (C) terms for genes differentially upregulated in HaCaT at -25°C relative to 37°C. The top 10 enriched GO BP (D) and MF (E) and reactome (F) terms for genes differentially upregulated in TIGK relative to HaCaT at -25°C relative to 37°C.

## Slide 6
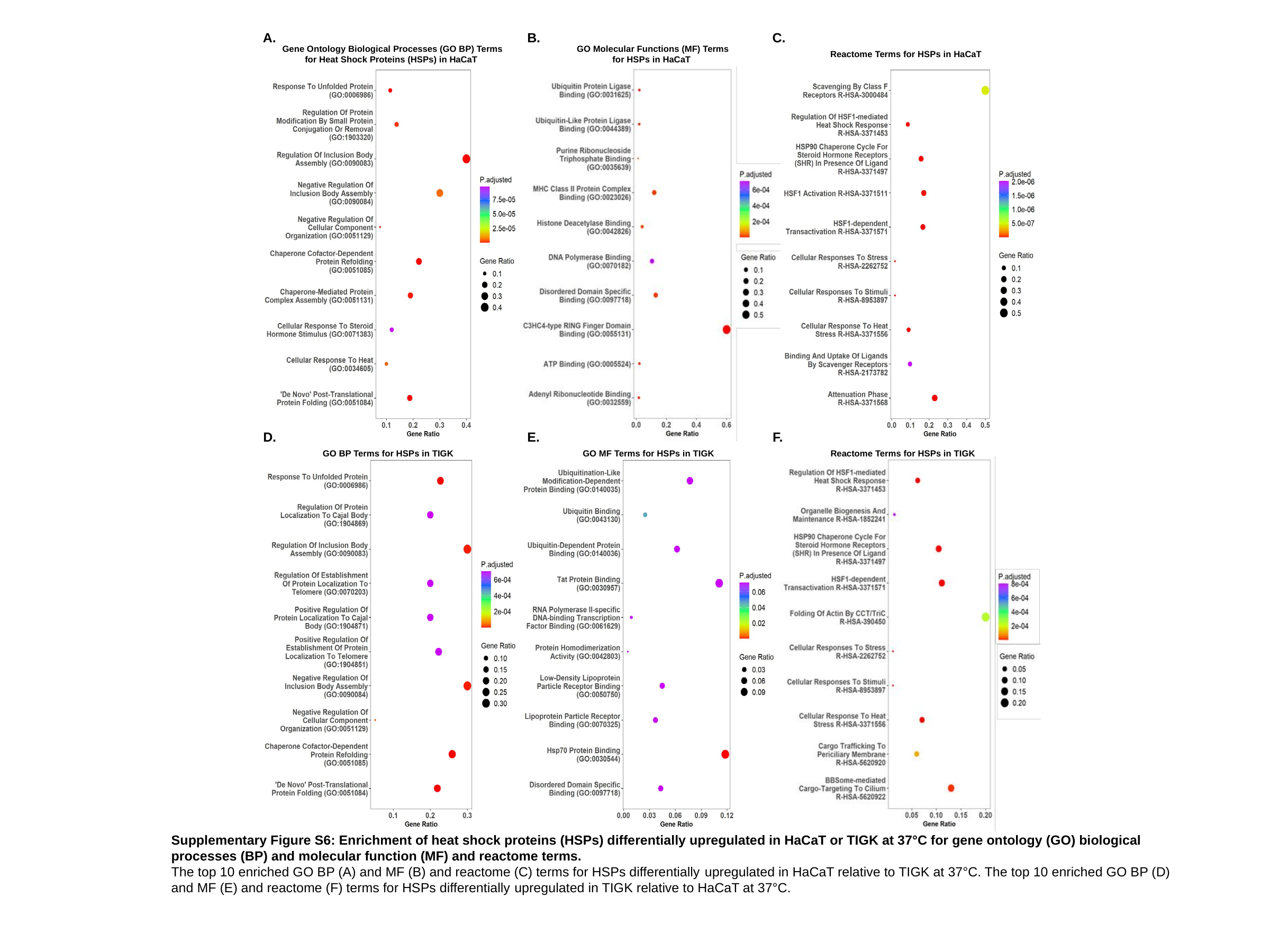

C.
A.
B.
Gene Ontology Biological Processes (GO BP) Terms for Heat Shock Proteins (HSPs) in HaCaT
GO Molecular Functions (MF) Terms for HSPs in HaCaT
Reactome Terms for HSPs in HaCaT
F.
D.
E.
GO BP Terms for HSPs in TIGK
GO MF Terms for HSPs in TIGK
Reactome Terms for HSPs in TIGK
Supplementary Figure S6: Enrichment of heat shock proteins (HSPs) differentially upregulated in HaCaT or TIGK at 37°C for gene ontology (GO) biological processes (BP) and molecular function (MF) and reactome terms.
The top 10 enriched GO BP (A) and MF (B) and reactome (C) terms for HSPs differentially upregulated in HaCaT relative to TIGK at 37°C. The top 10 enriched GO BP (D) and MF (E) and reactome (F) terms for HSPs differentially upregulated in TIGK relative to HaCaT at 37°C.

## Slide 7
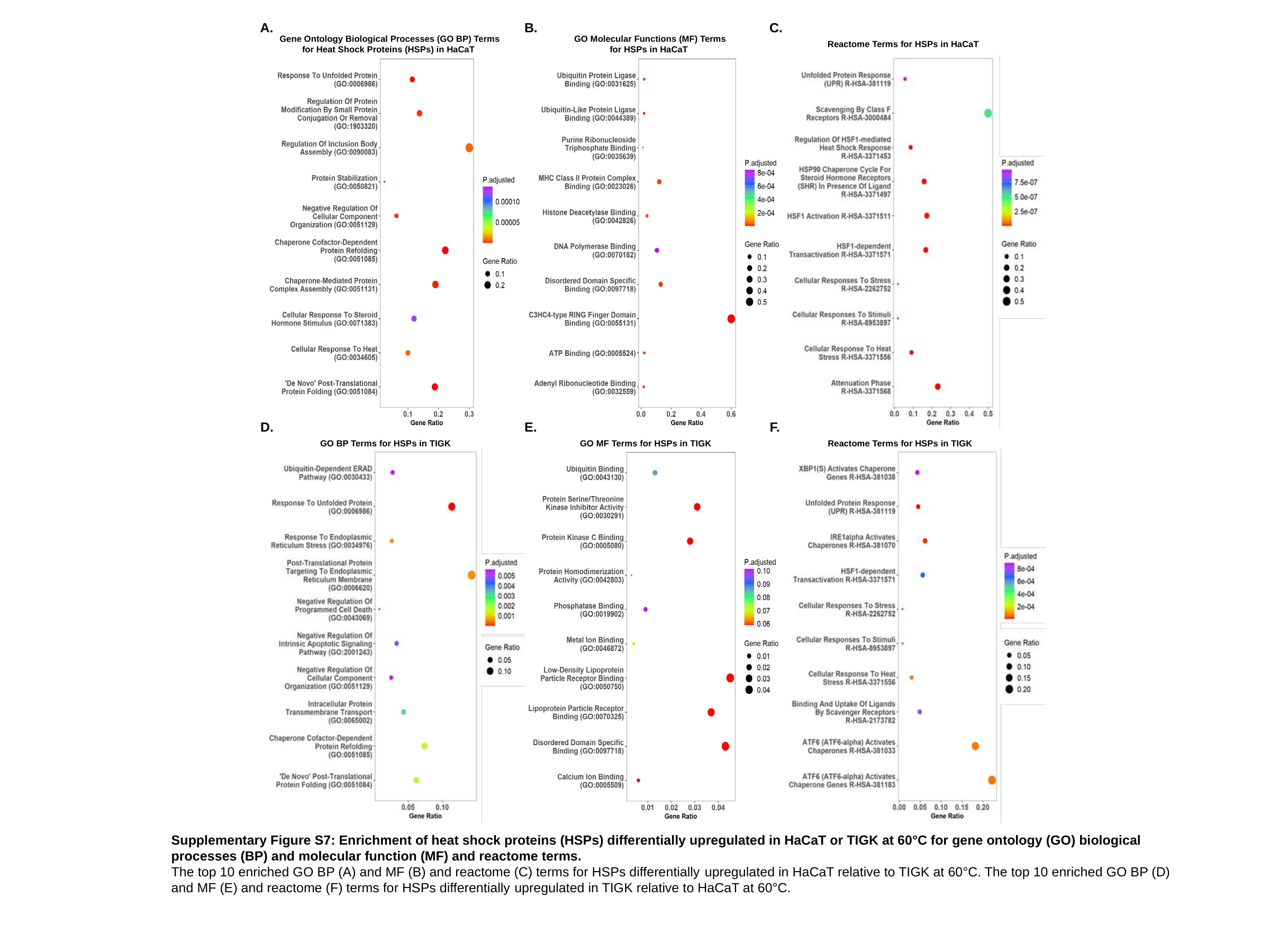

C.
A.
B.
Gene Ontology Biological Processes (GO BP) Terms for Heat Shock Proteins (HSPs) in HaCaT
GO Molecular Functions (MF) Terms for HSPs in HaCaT
Reactome Terms for HSPs in HaCaT
F.
D.
E.
GO BP Terms for HSPs in TIGK
GO MF Terms for HSPs in TIGK
Reactome Terms for HSPs in TIGK
Supplementary Figure S7: Enrichment of heat shock proteins (HSPs) differentially upregulated in HaCaT or TIGK at 60°C for gene ontology (GO) biological processes (BP) and molecular function (MF) and reactome terms.
The top 10 enriched GO BP (A) and MF (B) and reactome (C) terms for HSPs differentially upregulated in HaCaT relative to TIGK at 60°C. The top 10 enriched GO BP (D) and MF (E) and reactome (F) terms for HSPs differentially upregulated in TIGK relative to HaCaT at 60°C.

## Slide 8
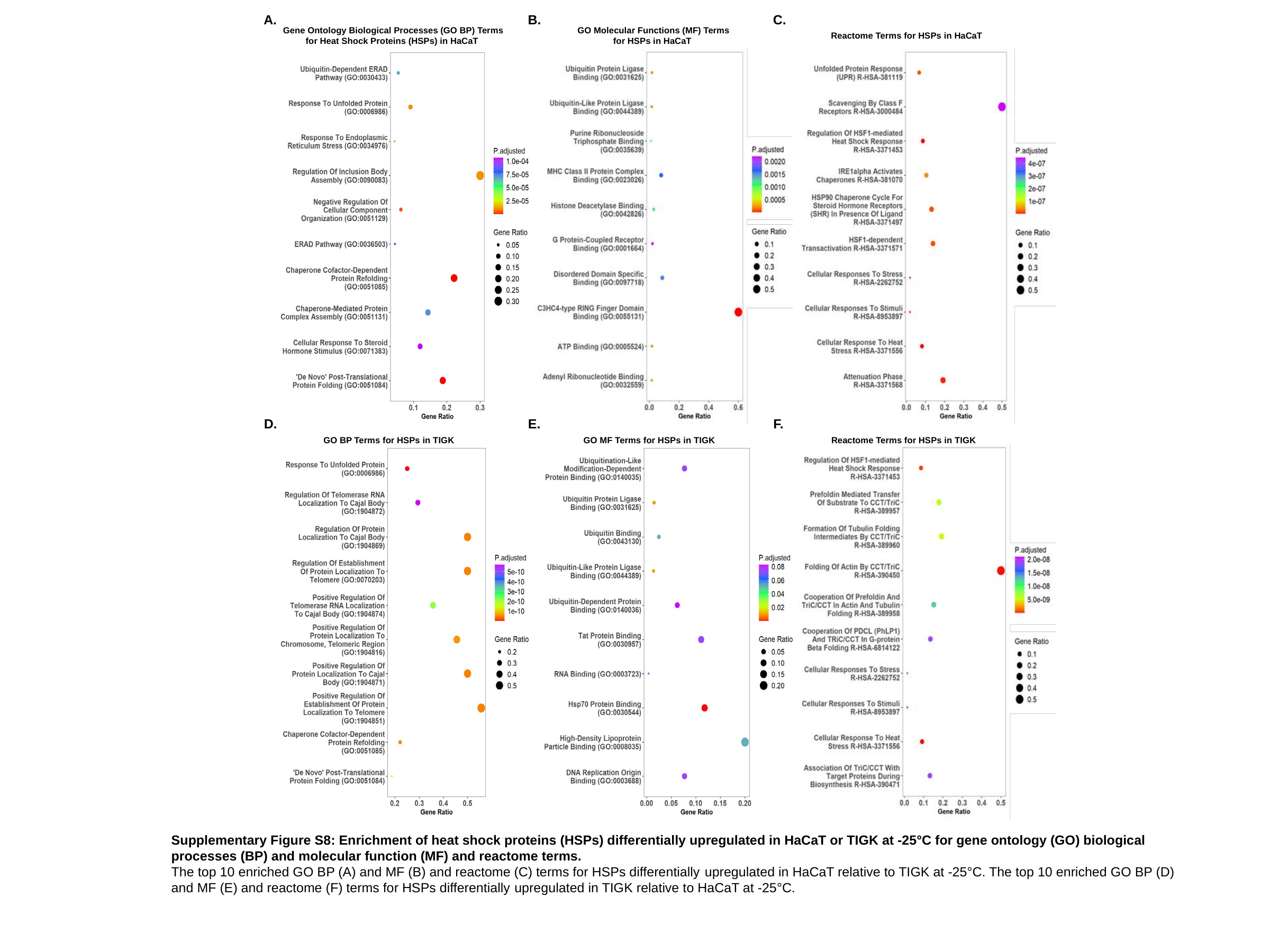

C.
A.
B.
Gene Ontology Biological Processes (GO BP) Terms for Heat Shock Proteins (HSPs) in HaCaT
GO Molecular Functions (MF) Terms for HSPs in HaCaT
Reactome Terms for HSPs in HaCaT
F.
D.
E.
GO BP Terms for HSPs in TIGK
GO MF Terms for HSPs in TIGK
Reactome Terms for HSPs in TIGK
Supplementary Figure S8: Enrichment of heat shock proteins (HSPs) differentially upregulated in HaCaT or TIGK at -25°C for gene ontology (GO) biological processes (BP) and molecular function (MF) and reactome terms.
The top 10 enriched GO BP (A) and MF (B) and reactome (C) terms for HSPs differentially upregulated in HaCaT relative to TIGK at -25°C. The top 10 enriched GO BP (D) and MF (E) and reactome (F) terms for HSPs differentially upregulated in TIGK relative to HaCaT at -25°C.

## Slide 9
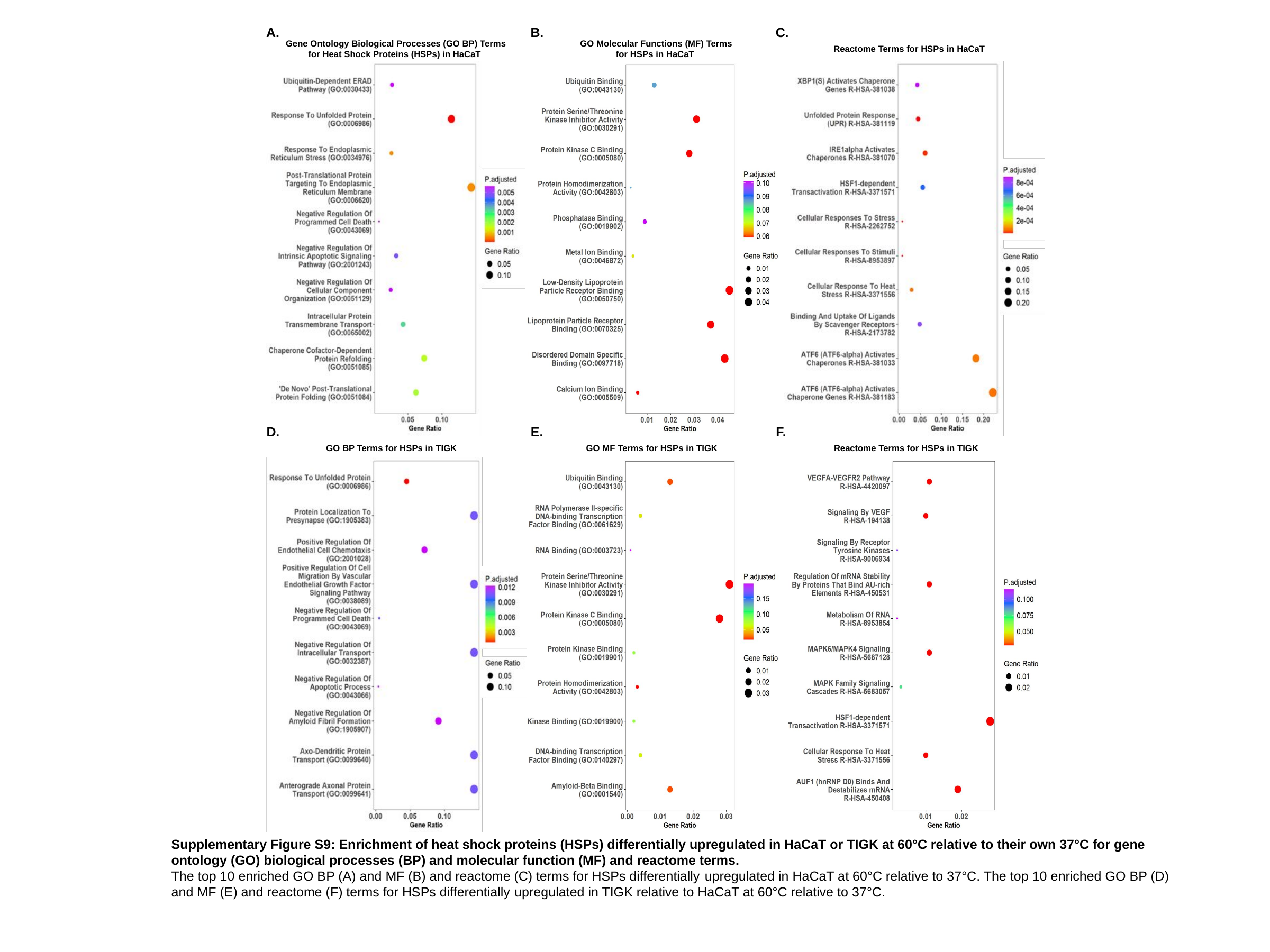

C.
A.
B.
Gene Ontology Biological Processes (GO BP) Terms for Heat Shock Proteins (HSPs) in HaCaT
GO Molecular Functions (MF) Terms for HSPs in HaCaT
Reactome Terms for HSPs in HaCaT
F.
D.
E.
GO BP Terms for HSPs in TIGK
GO MF Terms for HSPs in TIGK
Reactome Terms for HSPs in TIGK
Supplementary Figure S9: Enrichment of heat shock proteins (HSPs) differentially upregulated in HaCaT or TIGK at 60°C relative to their own 37°C for gene ontology (GO) biological processes (BP) and molecular function (MF) and reactome terms.
The top 10 enriched GO BP (A) and MF (B) and reactome (C) terms for HSPs differentially upregulated in HaCaT at 60°C relative to 37°C. The top 10 enriched GO BP (D) and MF (E) and reactome (F) terms for HSPs differentially upregulated in TIGK relative to HaCaT at 60°C relative to 37°C.

## Slide 10
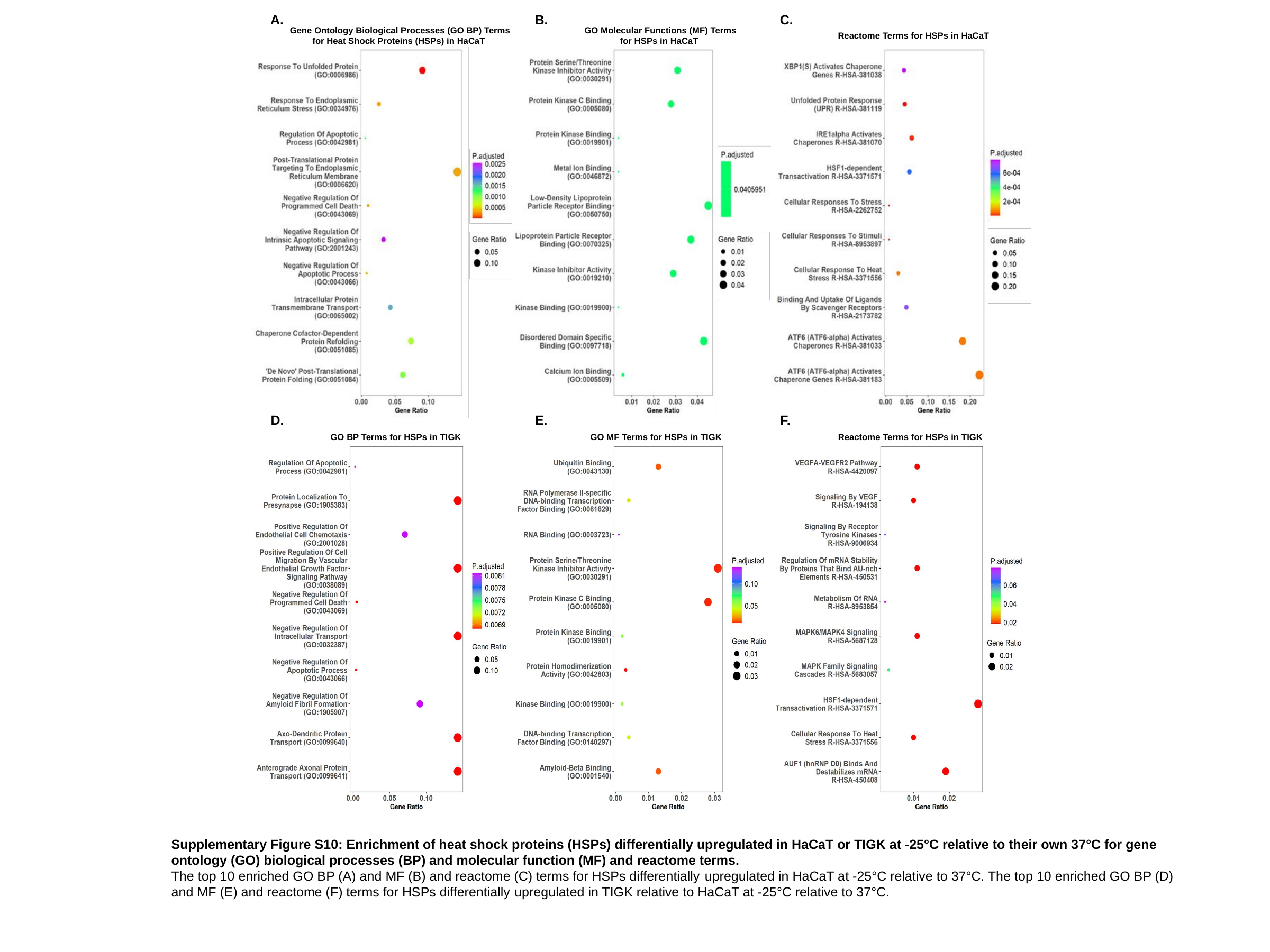

C.
A.
B.
Gene Ontology Biological Processes (GO BP) Terms for Heat Shock Proteins (HSPs) in HaCaT
GO Molecular Functions (MF) Terms for HSPs in HaCaT
Reactome Terms for HSPs in HaCaT
F.
D.
E.
GO BP Terms for HSPs in TIGK
GO MF Terms for HSPs in TIGK
Reactome Terms for HSPs in TIGK
Supplementary Figure S10: Enrichment of heat shock proteins (HSPs) differentially upregulated in HaCaT or TIGK at -25°C relative to their own 37°C for gene ontology (GO) biological processes (BP) and molecular function (MF) and reactome terms.
The top 10 enriched GO BP (A) and MF (B) and reactome (C) terms for HSPs differentially upregulated in HaCaT at -25°C relative to 37°C. The top 10 enriched GO BP (D) and MF (E) and reactome (F) terms for HSPs differentially upregulated in TIGK relative to HaCaT at -25°C relative to 37°C.

## Slide 11
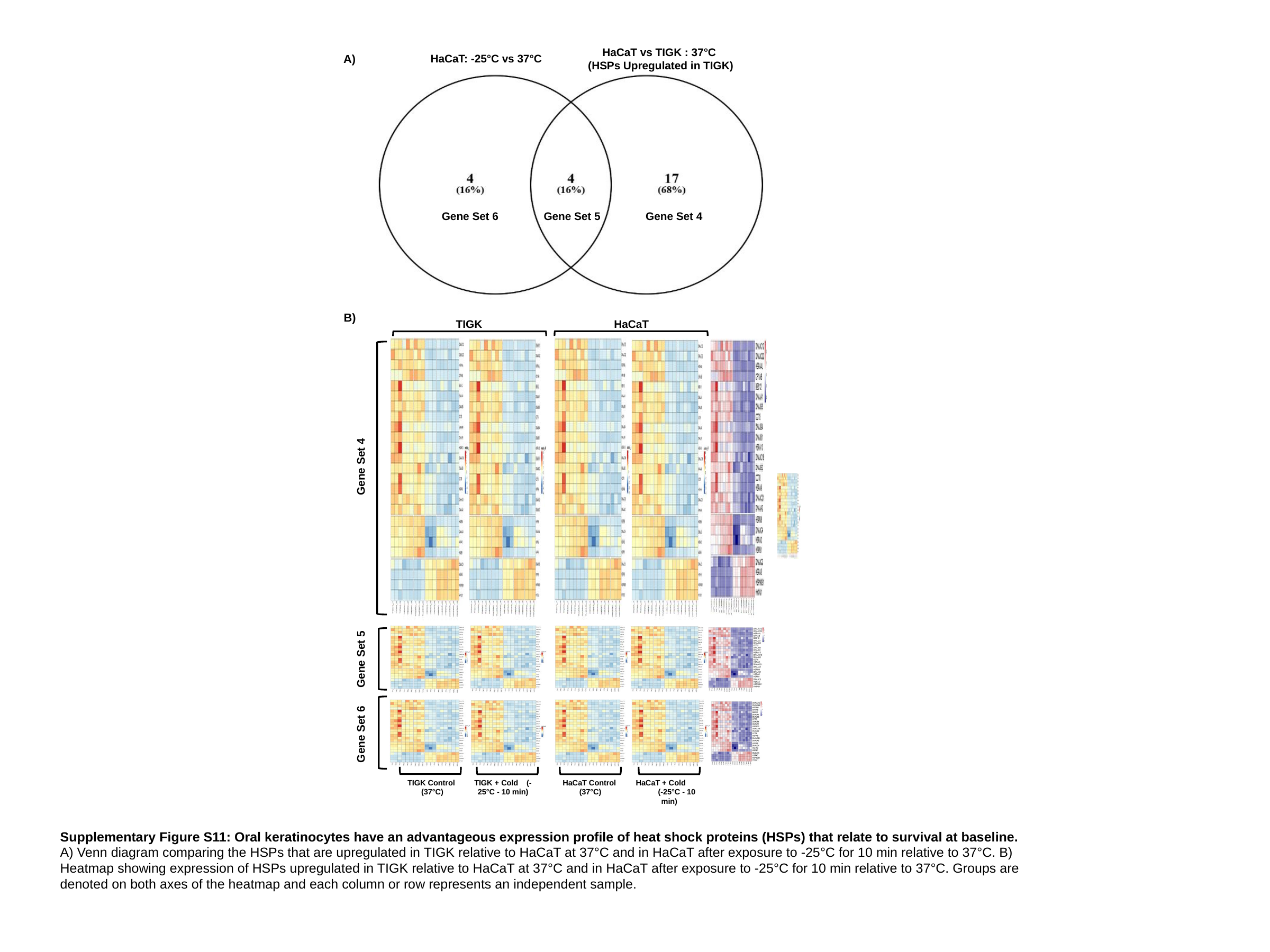

HaCaT vs TIGK : 37°C
(HSPs Upregulated in TIGK)
HaCaT: -25°C vs 37°C
Gene Set 6
Gene Set 5
Gene Set 4
A)
B)
TIGK
HaCaT
Gene Set 4
Gene Set 5
Gene Set 6
TIGK Control
(37°C)
TIGK + Cold (-25°C - 10 min)
HaCaT Control
(37°C)
HaCaT + Cold (-25°C - 10 min)
Supplementary Figure S11: Oral keratinocytes have an advantageous expression profile of heat shock proteins (HSPs) that relate to survival at baseline.
A) Venn diagram comparing the HSPs that are upregulated in TIGK relative to HaCaT at 37°C and in HaCaT after exposure to -25°C for 10 min relative to 37°C. B) Heatmap showing expression of HSPs upregulated in TIGK relative to HaCaT at 37°C and in HaCaT after exposure to -25°C for 10 min relative to 37°C. Groups are denoted on both axes of the heatmap and each column or row represents an independent sample.

## Slide 12
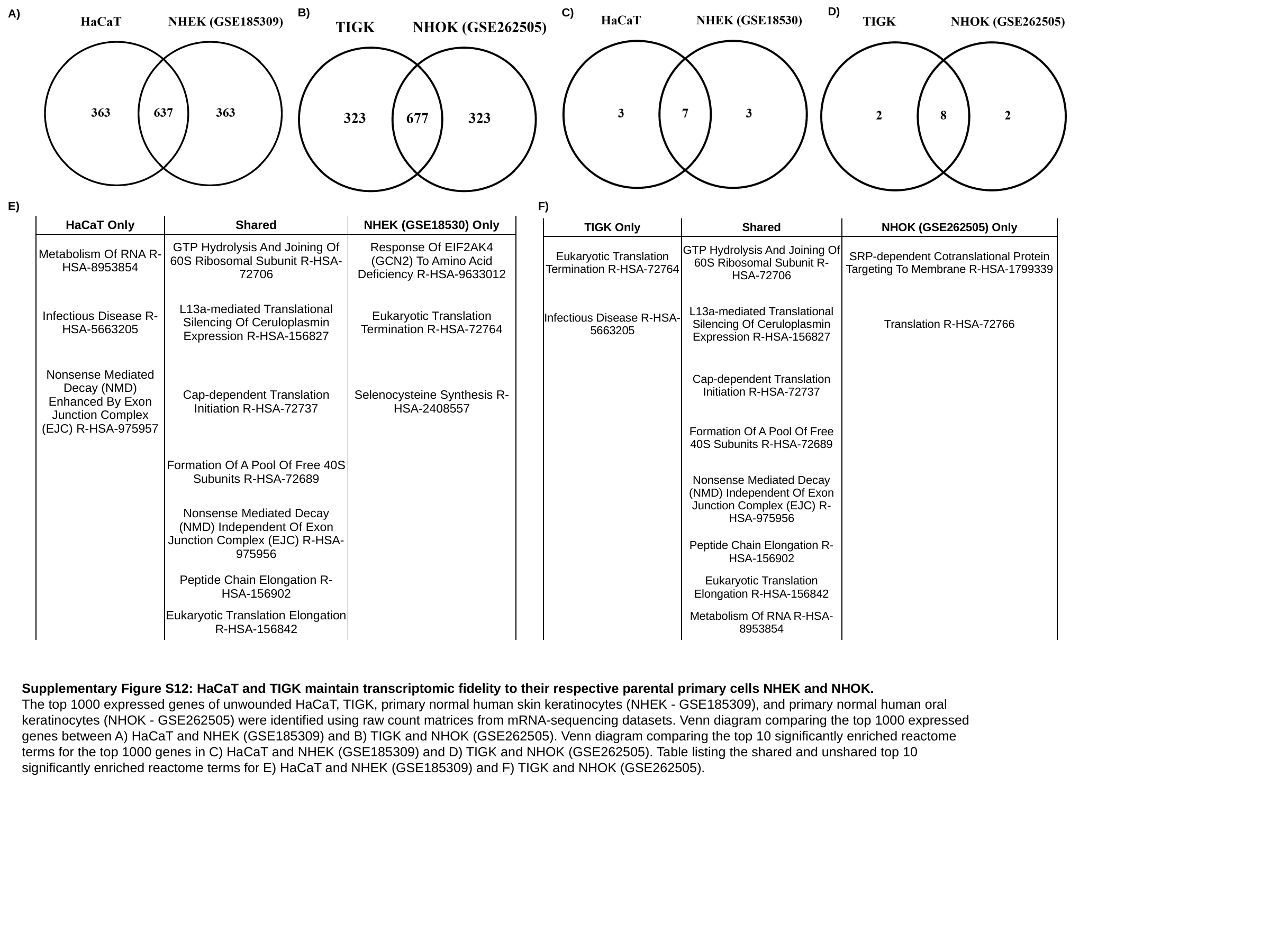

D)
B)
C)
A)
E)
F)
| HaCaT Only | Shared | NHEK (GSE18530) Only |
| --- | --- | --- |
| Metabolism Of RNA R-HSA-8953854 | GTP Hydrolysis And Joining Of 60S Ribosomal Subunit R-HSA-72706 | Response Of EIF2AK4 (GCN2) To Amino Acid Deficiency R-HSA-9633012 |
| Infectious Disease R-HSA-5663205 | L13a-mediated Translational Silencing Of Ceruloplasmin Expression R-HSA-156827 | Eukaryotic Translation Termination R-HSA-72764 |
| Nonsense Mediated Decay (NMD) Enhanced By Exon Junction Complex (EJC) R-HSA-975957 | Cap-dependent Translation Initiation R-HSA-72737 | Selenocysteine Synthesis R-HSA-2408557 |
| | Formation Of A Pool Of Free 40S Subunits R-HSA-72689 | |
| | Nonsense Mediated Decay (NMD) Independent Of Exon Junction Complex (EJC) R-HSA-975956 | |
| | Peptide Chain Elongation R-HSA-156902 | |
| | Eukaryotic Translation Elongation R-HSA-156842 | |
| TIGK Only | Shared | NHOK (GSE262505) Only |
| --- | --- | --- |
| Eukaryotic Translation Termination R-HSA-72764 | GTP Hydrolysis And Joining Of 60S Ribosomal Subunit R-HSA-72706 | SRP-dependent Cotranslational Protein Targeting To Membrane R-HSA-1799339 |
| Infectious Disease R-HSA-5663205 | L13a-mediated Translational Silencing Of Ceruloplasmin Expression R-HSA-156827 | Translation R-HSA-72766 |
| | Cap-dependent Translation Initiation R-HSA-72737 | |
| | Formation Of A Pool Of Free 40S Subunits R-HSA-72689 | |
| | Nonsense Mediated Decay (NMD) Independent Of Exon Junction Complex (EJC) R-HSA-975956 | |
| | Peptide Chain Elongation R-HSA-156902 | |
| | Eukaryotic Translation Elongation R-HSA-156842 | |
| | Metabolism Of RNA R-HSA-8953854 | |
Supplementary Figure S12: HaCaT and TIGK maintain transcriptomic fidelity to their respective parental primary cells NHEK and NHOK.
The top 1000 expressed genes of unwounded HaCaT, TIGK, primary normal human skin keratinocytes (NHEK - GSE185309), and primary normal human oral keratinocytes (NHOK - GSE262505) were identified using raw count matrices from mRNA-sequencing datasets. Venn diagram comparing the top 1000 expressed genes between A) HaCaT and NHEK (GSE185309) and B) TIGK and NHOK (GSE262505). Venn diagram comparing the top 10 significantly enriched reactome terms for the top 1000 genes in C) HaCaT and NHEK (GSE185309) and D) TIGK and NHOK (GSE262505). Table listing the shared and unshared top 10 significantly enriched reactome terms for E) HaCaT and NHEK (GSE185309) and F) TIGK and NHOK (GSE262505).
